# Epithelia-Sensory Neuron Cross Talk Underlies Cholestatic Itch Induced by Lysophosphatidylcholine

**DOI:** 10.1053/j.gastro.2021.03.049

**Published:** 2021-04-02

**Authors:** Yong Chen, Zi-Long Wang, Michele Yeo, Qiao-Juan Zhang, Ana E. López-Romero, Hui-Ping Ding, Xin Zhang, Qian Zeng, Sara L. Morales-Lázaro, Carlene Moore, Ying-Ai Jin, Huang-He Yang, Johannes Morstein, Andrey Bortsov, Marcin Krawczyk, Frank Lammert, Manal Abdelmalek, Anna Mae Diehl, Piotr Milkiewicz, Andreas E. Kremer, Jennifer Y. Zhang, Andrea Nackley, Tony E. Reeves, Mei-Chuan Ko, Ru-Rong Ji, Tamara Rosenbaum, Wolfgang Liedtke

**Affiliations:** 1 Department of Neurology, Duke University, Durham, North Carolina; 2 Center for Translational Pain Medicine, Department of Anesthesiology, Duke University, Durham, North Carolina; 3 Departamento de Neurociencia Cognitiva, Instituto de Fisiología Celular, Universidad Nacional Autónoma de México, Coyoacan, Mexico City, Mexico; 4 Department of Physiology and Pharmacology, Wake Forest University, Winston-Salem, North Carolina; 5 Department of Dermatology, Duke University, Durham, North Carolina; 6 Department of Biochemistry, Duke University, Durham, North Carolina; 7 Department of Neurobiology, Duke University, Durham, North Carolina; 8 Department of Chemistry, New York University, New York, New York; 9 Department of Medicine II, Saarland University Medical Center, Saarland University, Homburg, Germany; 10 Laboratory of Metabolic Liver Diseases, Center for Preclinical Research, Department of General, Transplant and Liver Surgery, Medical University of Warsaw, Warsaw, Poland; 11 Hannover Medical School MHH, Hannover, Germany; 12 Division of Gastroenterology, Department of Medicine, Duke University, Durham, North Carolina; 13 Liver and Internal Medicine Unit, Department of General, Transplant and Liver Surgery, Medical University of Warsaw, Warsaw, Poland; 14 Translation Medicine Group, Pomeranian Medical University, Szczecin, Poland; 15 Department of Medicine 1, Gastroenterology, Hepatology, Pneumology and Endocrinology, Friedrich-Alexander-University Erlangen-Nürnberg, Erlangen, Germany; 16 Department of Pathology, Duke University, Durham, North Carolina; 17 Department of Pharmacology and Cancer Biology, Duke University, Durham, North Carolina; 18 Department of Internal Medicine, Section on Molecular Medicine, Wake Forest School of Medicine, Winston-Salem, North Carolina; 19 Neurology Clinics for Headache, Head-Pain and Trigeminal Sensory Disorders, Duke University, Durham, North Carolina; 20 Clinics for Innovative Pain Therapy, Department of Anesthesiology, Duke University, Raleigh, North Carolina

**Keywords:** Lysophosphatidylcholine, Cholestatic Itch, Keratinocyte TRPV4, miR-146a, TRPV1 Pruriceptor

## Abstract

**BACKGROUND & AIMS:**

Limited understanding of pruritus mechanisms in cholestatic liver diseases hinders development of antipruritic treatments. Previous studies implicated lysophosphatidic acid (LPA) as a potential mediator of cholestatic pruritus.

**METHODS:**

Pruritogenicity of lysophosphatidylcholine (LPC), LPA’s precursor, was examined in naïve mice, cholestatic mice, and nonhuman primates. LPC’s pruritogenicity involving keratinocyte TRPV4 was studied using genetic and pharmacologic approaches, cultured keratinocytes, ion channel physiology, and structural computational modeling. Activation of pruriceptor sensory neurons by microRNA-146a (miR-146a), secreted from keratinocytes, was identified by in vitro and ex vivo Ca^2+^ imaging assays. Sera from patients with primary biliary cholangitis were used for measuring the levels of LPC and miR-146a.

**RESULTS:**

LPC was robustly pruritic in mice. TRPV4 in skin keratinocytes was essential for LPC-induced itch and itch in mice with cholestasis. Three-dimensional structural modeling, site-directed mutagenesis, and channel function analysis suggested a TRPV4 C-terminal motif for LPC binding and channel activation. In keratinocytes, TRPV4 activation by LPC induced extracellular release of miR-146a, which activated TRPV1^+^ sensory neurons to cause itch. LPC and miR-146a levels were both elevated in sera of patients with primary biliary cholangitis with itch and correlated with itch intensity. Moreover, LPC and miR-146a were also increased in sera of cholestatic mice and elicited itch in nonhuman primates.

**CONCLUSIONS:**

We identified LPC as a novel cholestatic pruritogen that induces itch through epithelia-sensory neuron cross talk, whereby it directly activates skin keratinocyte TRPV4, which rapidly releases miR-146a to activate skin-innervating TRPV1^+^ pruriceptor sensory neurons. Our findings support the new concept of the skin, as a sensory organ, playing a critical role in cholestatic itch, beyond liver, peripheral sensory neurons, and central neural pathways supporting pruriception.

## Graphical Abstract

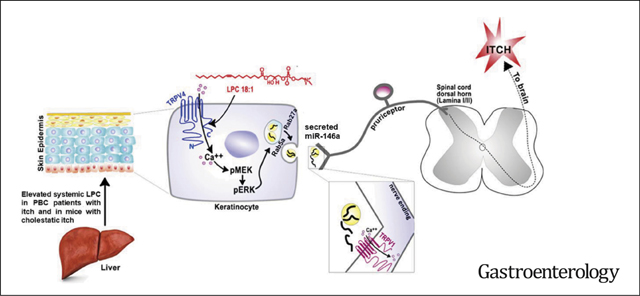

**T**reatment for itch (pruritus) associated with chronic liver disease represents a severe unmet medical need.^1^ Cholestatic itch is a debilitating symptom that has significant prevalence in patients with hepatobiliary diseases: namely, primary biliary cholangitis (PBC), primary sclerosing cholangitis, and intrahepatic cholestasis of pregnancy.^1^ Therapeutic recourse is dire, because the underlying pathophysiology remains largely elusive. Cholestatic itch has been linked to bile acids, bilirubin, progesterone metabolites, and lysophosphatidic acid (LPA).^2–6^ LPA is a bioactive phospholipid with diverse biological functions.^7^ Autotaxin catalyzes the hydrolysis of lysophosphatidylcholine (LPC) to LPA, and it has been demonstrated that levels of autotaxin and LPA correlate with itch intensity in patients with cholestatic liver disease.^2^ In addition, a recent study suggested that LPA induces itch via TRPV1 and TRPA1.^8^

Transient receptor potential (TRP) ion channels have been implicated in the molecular mechanisms of itch, with experimental studies supporting significant roles for the chemoirritant receptor TRPA1 and the heat-capsaicin receptor TRPV1 in primary sensory neurons, where both channels function not only in pain transduction but also in pruriception.^9^ TRPV4, a widely expressed multimodally activated channel expressed in both innervated epithelia and sensory neurons, has also been found relevant for experimental itch.^9,10^ Our group has demonstrated a novel propruritic role of TRPV4 in skin keratinocytes.^10^

LPC, the precursor of LPA, has been linked with a variety of diseases that are pruritic. For instance, previous studies demonstrated that LPC concentrations were increased in the blood^11^ and lesional skin^12^ of patients with psoriasis. Mass spectrometry of lesional skin from patients with atopic dermatitis revealed an increase of short-chain LPC species.^13^ Moreover, a recent study found that LPC concentrations were significantly elevated systemically in patients with uremia and pruritus.^14^ This evidence and the involvement of the LPC metabolite LPA in cholestatic itch prompted us to ask whether LPC could also contribute to cholestatic itch. We sought to address:

1. Does LPC induce itch?
2. Does LPC-triggered itch involve TRPV4 in skin keratinocytes, given the propruritic role of TRPV4 in these cells?
3. Does direct cross talk between skin keratinocytes andsensory neurons underlie LPC-induced itch?
4. Is LPC elevated systemically in cholestatic liver disease in experimental animals as well as in patients with itch?

Here we identified LPC as a novel pruritogen that elicited robust scratching behaviors in mice and nonhuman primates. LPC functioned via TPRV4 activation in keratinocytes, which we explain mechanistically in an LPC-TRPV4 binding model. Interestingly, this signaling in keratinocytes induced extracellular release of microRNA-146a (miR146a), which rapidly activated TRPV1^+^ pruriceptor sensory neurons to cause itch. Moreover, we found elevated levels of systemic LPC and miR-146a in patients with PBC with itch and also in cholestatic mice. In cholestatic mice, we observed dependence of scratching behavior and systemic concentration of miR-146a on keratinocyte TRPV4. Our findings suggest a hitherto underappreciated function of the epidermis and of TRPV4 in keratinocytes as a key signaling molecule in cholestatic itch.

## Materials and Methods

Additional details are provided in the Supplementary Materials.

### Animals

Wild-type (WT), C57bl/6j), *Trpv1*, *Trpa1*, and *Tlr7* knockout (KO) mice were from The Jackson Laboratory (Bar Harbor, ME). *Trpv4* KO mice were generated in our laboratory.^10^ PirtGCaMP3 mice, expressing the calcium indicator GCaMP3 in >96% of sensory neurons in the dorsal root ganglion (DRG), were from Dr Xinzhong Dong.^15^ Keratinocyte-specific, tamoxifen (tam)-inducible *Trpv4* KO (K14-Cre-ER^tam^::*Trpv4*^lox/lox^) mice were generated as previously described.^10^ Sensory neuron-specific *Trpv4* KO mice (Nav1.8-Cre::*Trpv4*^lox/lox^) were generated by mating *Trpv4*^*fl/fl*^ mice with Nav1.8-Cre mice. The *Cre* mice enable gene recombination commencing at birth selectively in sensory neurons expressing Nav1.8. Mice with inducible expression of constitutively active B-raf (V600E) in keratinocytes were generated by crossing B-raf^CA/+^ mice with K5-cre-ER^tam^ mice. All mouse lines have the C57bl/6 background. Only male mice (2–3 months) were used for in vivo behavioral assays. Male and female rhesus monkeys (*Macaca mulatta*, 11–18 years) were used for the scratching behavior study. All animal protocols were approved by the Institutional Animal Care and Use Committee, Duke University and Wake Forest University.

### Human Participants

Patients with PBC were recruited at Warsaw Medical University, Poland, and the University Hospital of Erlangen, Germany. Itch intensity was quantified at the blood draw using a visual analogue scale ranging from 0 to 10 (0–3: no/mild itch, 3–6: moderate itch, 6–10: severe/worst imaginable itch). Study protocols were approved by the local medical Institutional Review Boards.

### Behavioral Assessment

After an intradermal (ID) injection of 50 *μ*L of chemical solutions into the mouse dorsal neck, scratching behavior was recorded and quantified as described in a blinded way.^10^ To investigate the effects of the selective inhibitors on scratching behaviors induced by LPA, LPC, or miR-146a, mice received an intraperitoneal injection of 0.25 mL or an intrathecal (IT, see approach below) injection of 5 *μ*L of inhibitor solutions 15 minutes before pruritogen injections. To test whether LPC induces scratching behavior at the spinal cord level, 5 *μ*L of LPC was IT injected into the L4/L5 subarachnoid space. To examine whether ID injection of LPC or miR146a induces pain-like behavior, a mouse cheek model was used to differentiate itch from pain. To examine whether TRPV1-expressing sensory neurons contribute to itch induced by LPC-or miR-146a, we ablated the central terminals of TRPV1-expressing neurons by an IT injection of 200 ng resiniferatoxin.

The procedure to induce scratching behaviors in monkeys was performed as previously described.^16^ A total of 20 *μ*L of histamine, LPC, or miR-146a solution was ID injected into the hind limbs, and the scratching behavior was recorded.

A mouse model of cholestasis was induced by *α*-naphthyl isothiocyanate (ANIT) administration via oral gavage for 5 days at 25 mg/kg.^5^ The scratching behavior was recorded for 1 hour before daily ANIT treatment.

### In Vitro and Ex Vivo Ca^2+^ Imaging

Routine procedures were followed for Ca^2+^ imaging in cultured DRG neurons, keratinocytes, and human embryonic kidney (HEK) cells.^10^ Ca^2+^ imaging was conducted after loading with Fura2-AM (Invitrogen, Carlsbad, CA). A previously established method was followed for ex vivo Ca^2+^ imaging of DRG explants.^15^ Intact DRGs (L4 or L5) were isolated from naïve male or female Pirt-GCaMP3 mice. The explants were placed in preoxygenated artificial cerebrospinal fluid and imaged using a Zeiss-780 upright confocal microscope (Carl Zeiss, Oberkochen, Germany). To investigate the effects of the inhibitors on Ca^2+^ influx induced by LPA, LPC, or miR-146a (as indicated in Figure legends), cells or DRGs were incubated with the inhibitors for 15 minutes before stimulation.

### Electrophysiology

Currents were recorded using the inside-out configuration of the patch-clamp technique.^17^ Single-channel recordings were performed as described previously.^18^

### Western Blot, Immunohistochemistry, and Quantitative Real-Time Polymerase Chain Reaction

Experiments were performed according to the manufacturer’s instructions and the standard procedures, as previously described.^10^

### Measurement of Released Vesicles and Extracellular MicroRNA-146a From Cultured Keratinocytes or Sera

The supernatant of the cultured keratinocytes was harvested 15 minutes after LPC stimulation, followed by purification using a Vesicular Isolation kit (Invitrogen). Total RNA extraction was then performed using a Total RNA Isolation kit (Invitrogen). For human PBC sera or sera from ANIT-treated mice, RNA was isolated using miRNeasy Plasma/Serum kit (Qiagen, Valencia, CA). Complementary DNA synthesis from extracted RNAs was performed, and quantitative polymerase chain reactions for each sample were run in triplicates. To investigate the effects of the inhibitors on LPC-induced extracellular release of miR-146a (as indicated in Figure legends), cells were incubated with the inhibitors for 15 minutes before stimulation.

Vesicular release from cultured keratinocytes was quantified by detecting acetylcholinesterase activity in the extracellular release fluid.^19^

### Lysophosphatidylcholine Measurement in Sera and Skin

Blood and dorsal neck skin (∼0.5 × 0.5 cm) were harvested from ANIT- or control-treated mice at day 5. Total levels of LPC were determined by an enzymatic colorimetric method.^20^ Total level of LPC was detected at w1 mmol/L in sera of control mice.

Serum levels of LPC in patients with PBC were determined by the AbsoluteIDQ p180 kit (Biocrates Life Sciences AG, Innsbruck, Austria). The assay allows simultaneous quantification of 188 metabolites, including 14 species of LPC. Total level of these LPC species was ∼155 *μ*mol/L in patients with PBC without itch.

## Statistical Analysis

All data are expressed as mean ± standard error of the mean. The 2-tailed *t* test and 1-way or 2-way analysis of variance, followed by Tukey’s post hoc test, were used for group comparison and calculated with SPSS 25 software (IBM, Armonk, NY). For scratching behavior in nonhuman primates, analyses of repeated measures data were performed using a linear mixed model as implemented in SAS 9.4 software (SAS Institute Inc, Cary, NC). The correlated nature of repeated measures was taken into account by using an autoregressive correlation matrix in the model specification. Between-group contrasts were evaluated for statistical significance using an *F* statistic. Pearson’s correlation coefficient and the corresponding *P* value were calculated to assess the correlation between itch intensity and LPC and miR146a levels. *P* < .05 indicated statistically significant differences.

## Results

### Lysophosphatidylcholine Induces Itch via Keratinocyte TRPV4

**Figure 1.**
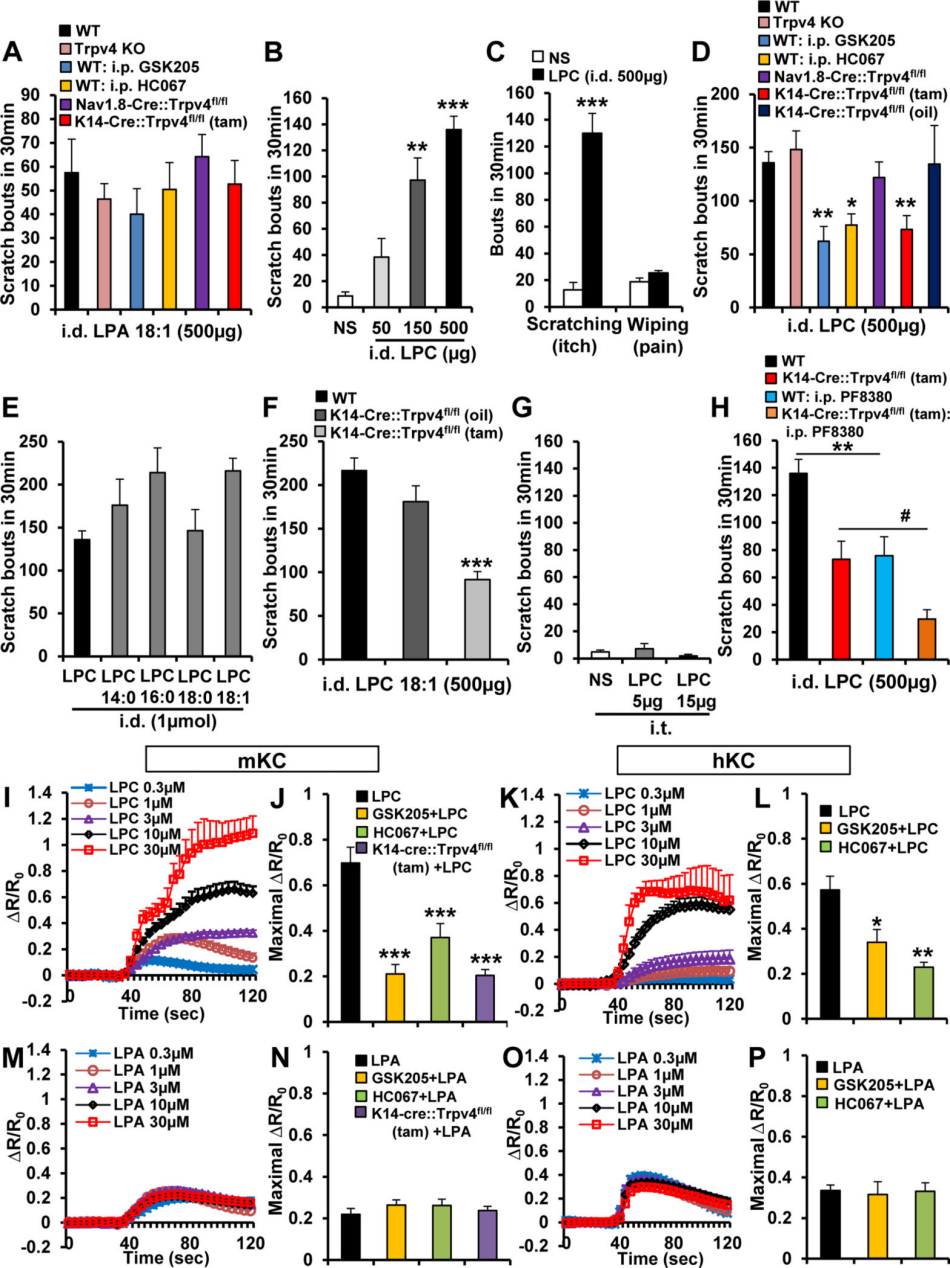
Scratching behavior induced by LPC, but not LPA, requires *Trpv4* in skin keratinocytes. (*A*) LPA(18:1)-induced itch was not significantly altered in mice: *Trpv4* KO, WT intraperitoneally (i.p.) pretreated with TRPV4 inhibitors GSK205 (20 mg/kg) or HC067047 (20 mg/kg), sensory neuron-*Trpv4* cKO (Nav1.8-Cre::Trpv4^fl/fl^), or keratinocyte-*Trpv4* cKO (K14-Cre::Trpv4^fl/fl^, tam-inducible (n = 4–7 mice/group). i.d., intradermal. (*B*) LPC (egg-LPC) elicited dose-dependent scratching. **P < .01 and ***P < .001 vs normal saline (NS) (n = 5 mice for NS and 50 *μ*g, 6 for 150 *μ*g, and 11 for 500 *μ*g). *(C*) Note LPC induced robust scratching (itch), not wiping (pain) response in the mouse cheek model. ***P < .001 vs NS (n = 4–5 mice/group). (*D*) LPC induced itch that was not significantly attenuated in *Trpv4* KO mice or in sensory neuron-*Trpv4* cKO mice but was significantly reduced in WT mice i.p. pretreated with GSK205 (20 mg/kg) or HC067 (20 mg/kg) and in keratinocyte-*Trpv4* cKOs. *P < .05 and **P < .01 vs WT LPC (n = 7–11 mice/group). (*E*) Different species of LPC also evoked robust scratching, with LPC(18:1) most potent (n = 5 mice/species except n = 11 for LPC). (*F*) LPC(18:1)-evoked scratching was also significantly reduced in keratinocyte-*Trpv4* cKO mice. ***P < .001 vs WT (n = 4–5 mice/group). (*G*) Intrathecal (i.t.) injection of LPC did not elicit scratching (n = 5 mice/group). (*H*) LPC-induced itch was attenuated in WT mice by i.p. pretreatment with autotaxin inhibitor PF8380 (10 mg/kg), and this attenuation was further augmented in keratinocyte-*Trpv4* cKO mice. **P < .01 and #*P* < .05 (n = 8–11 mice/group). (*I–L*) LPC-induced Ca^2+^ influx in a dose-dependent manner in (*I*) mouse keratinocytes (mKCs) and (*K*) human keratinocytes (hKCs). R/R_0_ indicates the fraction of the increase of the ratio over the baseline ratio divided by baseline ratio. (*J* and *L*) LPC-induced Ca^2+^ influx was significantly reduced by GSK205 or HC067047 (10 *μ*mol/L) and in (*J*) KC from KCTrpv4 cKO mice. *P < .05, **P < .01 and ***P < .001 vs LPC (n ≥ 180 cells recorded/treatment). (*M–P*) LPA(18:1)-induced Ca^2+^ influx was not dose-dependent and was less robust than that of LPC in (*M*) mouse and (*O*) human KCs. In addition, LPA (10 *μ*mol/L)-induced Ca^2+^ signal remained unchanged with (*N* and *P*) GSK205 or HC067047 pretreatment (10 *μ*mol/L) or in (*N*) keratinocytes from keratinocyte-*Trpv4* cKO mice (n ≥ 200 cells recorded/treatment). Two-tailed *t* test for *C* and 1-way analysis of variance with Tukey’s post hoc test for the rest. The *error bars* show the standard error of the mean.

First, we addressed whether the known cholestatic pruritogen, LPA, induces pruritus via TRPV4. We found no evidence that LPA’s pruritogenicity relies on TRPV4 (Figure 1A, Supplementary Figure 1).

Next we examined pruritogenicity of LPC, the direct metabolic precursor of LPA. In mice, we observed dose-dependent scratching evoked by LPC (Figure 1B). LPC was robustly more potent than LPA, eliciting scratching but not pain-related wiping behavior (Figure 1C). Next, we examined TRPV4-dependence of LPC-induced scratching. Whereas WT and pan-null *Trpv4* KO mice did not differ, mice treated systemically with TRPV4 inhibitors, GSK205 and HC067047, showed significantly reduced LPC-induced scratching (Figure 1D). We reasoned that findings of acute *TRPV4* inhibition attenuating LPC-induced scratching suggest *TRPV4* dependence, whereas the role of *Trpv4* was masked in pan-null *Trpv4* KO mice likely because of gene-regulatory developmental compensation. To resolve this issue, we investigated LPC-induced scratching in *Trpv4* conditional KO (cKO) mice. Tamoxifen-induced keratinocyte-*Trpv4* cKO mice (K14-Cre-ER^tam^::*Trpv4*^fl/fl^) showed >50% reduction, whereas sensory neuron-*Trpv4* cKO mice (Nav1.8-Cre::*Trpv4*^fl/fl^) did not differ compared with controls (Figure 1D). These findings indicate that skin keratinocyte-TRPV4, not sensory neuron-TRPV4, is needed for LPC-induced scratching. Different species of LPC induced robust scratching, with LPC(18:1) the most potent (Figure 1E). Moreover, LPC(18:1)-induced itch was also reduced in keratinocyte-*Trpv4* cKO mice (Figure 1F). With clear-cut findings that ID LPC evokes robust scratching and relies on keratinocyte-*Trpv4*, we sought to elucidate whether selective LPC stimulation of peripheral sensory neurons evokes sensory behaviors. We demonstrated that IT LPC did not elicit itch (Figure 1G) but rather long-lasting pain relying on sensory neuron-*Trpv4* (Supplementary Figure 2).These results suggest that LPC intradermally elicits itch via a nonneuronal peripheral mechanism critically involving keratinocyte-TRPV4, whereas LPC intrathecally relies on sensory neuron-TRPV4 to evoke pain.

Next, we tested whether the LPC/LPA conversion contributes to LPC’s pruritogenicity. If so, we expected the attenuated LPC-caused scratching in keratinocyte-*Trpv4* cKO mice to be suppressed further when inhibiting the LPC→LPA conversion, which would lead to absence of LPA as metabolic product from LPC, and presence of LPC only. In that case the effect of selective knockdown of LPC’s molecular target in keratinocytes, TRPV4, would have more impact, which means less scratching. Indeed, using the selective autotaxin-inhibitor PF8380, we observed significant attenuation of residual LPC-induced scratching in keratinocyte-*Trpv4* cKO mice. These findings suggest (1) the LPC→LPA conversion is relevant for scratching in vivo, (2) LPC, but not its “co-pruritogen” metabolite LPA, evokes scratching directly via keratinocyte-TRPV4 (Figure 1H), and (3) that residual itch is, at least partially, mediated via LPA and itch-relevant signaling of LPA to LPA-R5 and the TRPV1 pruriceptor.^8^

We next determined whether LPC activatesTRPV4 in skin keratinocytes. We noticed that LPC, but not LPA, triggered Ca^2+^ influx in cultured mouse and human keratinocytes in a TRPV4-dependent manner (Figure 1I–P).

We generated several relevant negative results (Supplementary Figures 3 and 4). LPC-induced Ca^2+^ influx was independent of TRPV3, which is abundantly expressed in skin keratinocytes and known to contribute to itch.^21^ In addition, LPC-induced itch was not significantly altered when inhibiting TRPV3, indicating the pruritogenic effects of LPC may not rely on TRPV3. Similar findings were recorded for the mechanosensitive-channel Piezo1 in keratinocytes^22^ and for pruritogenic effects of Piezo1 activation using Yoda-1, which were independent of TRPV4.

### Lysophosphatidylcholine Activates TRPV4 Directly via a Putative C-Terminal Binding Pocket

We next tested whether LPC activates TRPV4 by direct binding to the channel. LPC(18:1), a major bioactive LPC subspecies, was selected because it is the most potent pruritogen (Figure 1E).

We first found there were no significant effects of the inhibition of G*α*_q_, phospholipase C, and G*β*_γ_ on LPC(18:1)induced Ca^2+^ signals (Supplementary Figure 5), suggesting LPC does not activate TRPV4 via G-protein-coupled receptor signaling.

**Figure 2.**
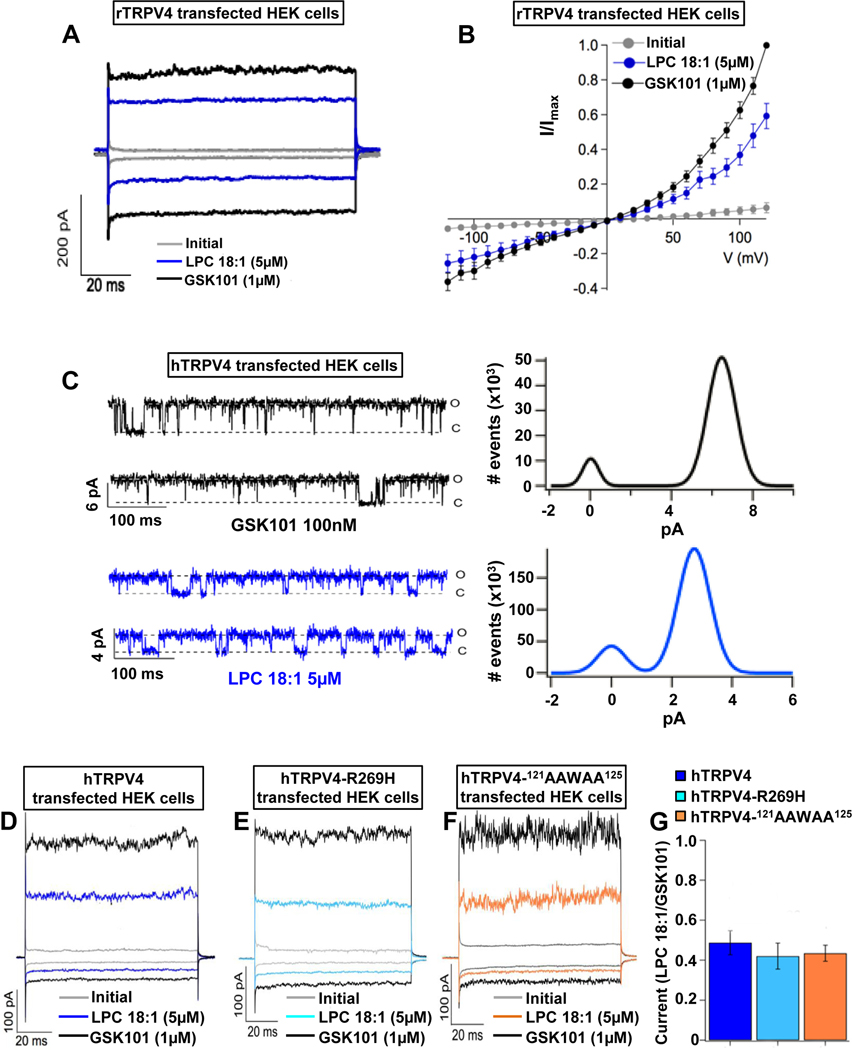
LPC directly activates TRPV4 channels. (*A* and *B*) Representative current-time plots from excised inside-out membrane-patches in rTRPV4-transfected HEK cells. These were obtained initially without agonist (*gray*), in the presence of LPC(18:1) (*blue*) or TRPV4 agonist GSK1016790A (GSK101; *black*). (*A*) Traces shown were recorded at −60 and +60 mV. (*B*) Current-voltage relationships from −120 to +120 mV (n = 5 cells/condition). (*C*) Single-channel recordings (*left*) of hTRPV4 activated with GSK101 or LPC(18:1) at +60 mV (o, open; c, closed state of TRPV4); all-point histograms were obtained from the traces (*right*). The average for the open level amplitudes was 6.45 ± 0.55 pA with an open probability of 0.85 ± 0.08 for GSK101 and 3.27 ± 0.41 pA and 0.75 ± 0.08 for LPC(18:1) (n = cells/condition). (*D–G*) Activation of hTRPV4 by LPC(18:1) remained unchanged with phosphatidylinositol 4,5-bisphosphate interaction mutations. Representative currents for (*D*) hTRPV4(WT), (*E*) hTRPV4(R269H), and (*F*) hTRPV4(^121^AAWAA^125^) channels. Currents were obtained initially without agonist (*gray*), in the presence of LPC(18:1) (*blue*, *sky blue*, and *orange*, respectively) or GSK101 (*black*) at −60 and +60 mV. (G) There were no significant differences in currents among hTRPV4(WT), hTRPV4(R269H), and hTRPV4(^121^AAWAA^125^) in response to LPC(18:1). Data were normalized to activation with GSK101 (n = 5–6 cells/condition) and analyzed by 1-way analysis of variance with Tukey’s post hoc test. The error bars indicate the standard error the mean.

We then conducted patch-clamp recordings of HEK293 cells overexpressing rat (r)TRPV4 and human (h)TRPV4, using the inside-out configuration, and demonstrated that LPC(18:1) activated TRPV4 at 54% of control currents evoked by the TRPV4-selective agonist GSK101 (Figure 2A and B, Supplementary Figure 6A). We recorded a relevant underlying channel physiologic metric by conducting single-channel recordings in hTRPV4-expressig HEK293 cells; namely, that activation of TRPV4 by LPC(18:1) accounted for ∼50% of the GSK101-evoked current (Figure 2C).

Based on previous observations that LPA interacts with the phosphatidylinositol 4,5-bisphosphate (PIP_2_) binding site of TRPV1,^23^ we also tested whether hTRPV4 with mutations in PIP_2_ interaction sites affect activation by LPC(18:1). TRPV4 channels with R269H^24^ and^121^ AAWAA^125^ mutations^25^ responded similarly as hTRPV4(WT) channels, demonstrating that these sites are not required for activation by LPC(18:1) (Figure 2D–G).

**Figure 3.**
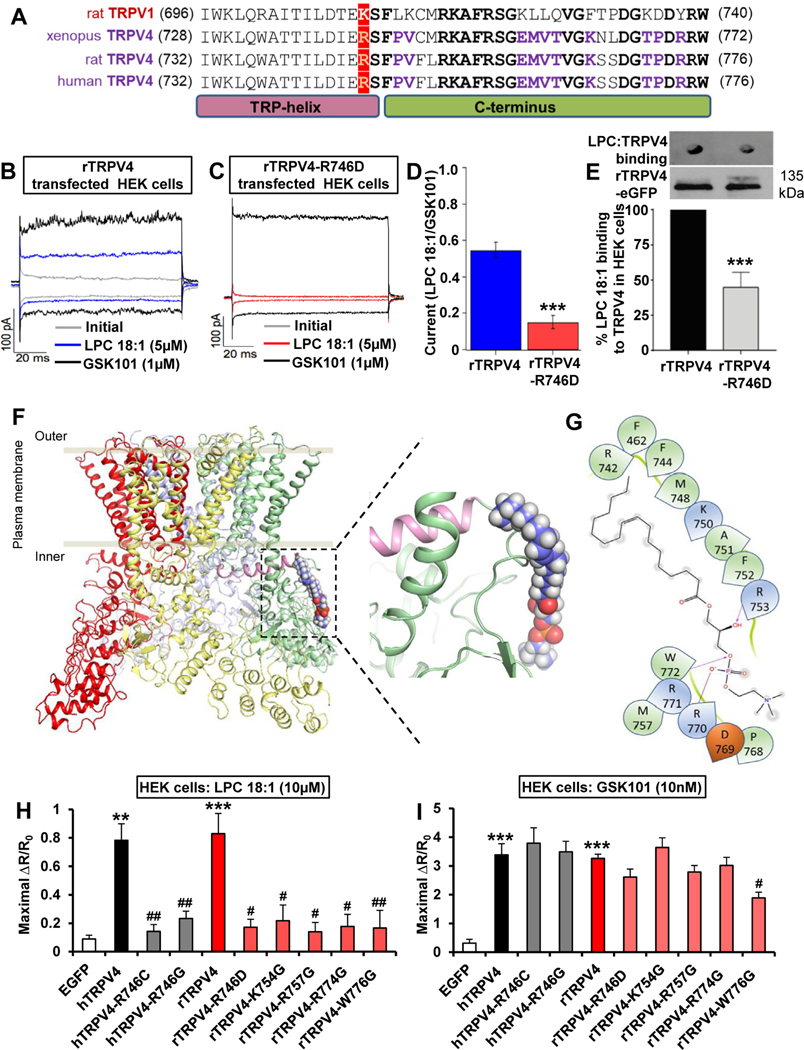
LPC activates TRPV4 directly via a C-terminal binding pocket. (*A*) Sequence alignment of the C-terminus comprising the TRP helix of rTRPV1, *Xenopus* TRPV4, rTRPV4, and hTRPV4. Note conservation of positive charge at position K710 for TRPV1, R742 for *Xenopus* TRPV4, and R746 for rTRPV4 and hTRPV4 (in *red*). Identical residues, shared between TRPV1 and TRPV4, C-terminal of this key residue are bolded in black. Identical residues, conserved only in vertebrate TRPV4, C-terminal to R742/R746 are bolded in *purple*. (*B–E*) Representative currents from excised inside-out membrane patches in (*B*) rTRPV4-transfected HEK cells or (*C*) rTRPV4(R746D) were obtained without agonist stimulation (*gray*), in the presence of LPC(18:1) (*blue, red*) or TRPV4 agonist GSK1016790A (GSK101, *black*). Traces shown were obtained at −60 and +60 mV. (*D*) There was a significant current reduction in rTRPV4(R746D)-transfected HEK cells when activated with LPC(18:1). Data were normalized to activation with GSK101. ***P < .001 vs rTRPV4 (n = 5 cells/group). (*E*) In vitro interaction assays show significantly reduced binding of LPC(18:1) to rTRPV4(R746D). eGFP, enhanced green fluorescent protein. ***P < .001 vs rTRPV4 (n = 3 assays/ group). (*F* and *G*) Based on alignment and established TRPV4 structure (crystal, cryo-electron microscopy), derived from *Xenopus tropicalis* TRPV4, note our structural model that explains binding of LPC(18:1) to a series of positively charged AA750–772; with R742 as a postulated structural determinant of this binding. The *left* rendering shows the TRPV4 tetramer (each subunit is in a different color) as it integrates into the plasma membrane, with the green subunit binding of LPC(18:1). The *right schematic* shows binding of LPC(18:1) to the TRPV4 C-terminus at higher resolution. (*H* and *I*) The Ca^2+^ signal induced by LPC(18:1) was drastically reduced in TRPV4-transfected HEK cells (TRPV4 mutations R746C, R746G, R746D, K754G, R757G, R774G, and W776G). In contrast, the GSK101-induced Ca^2+^ signal was not significantly disrupted, except a moderate reduction with mutation W776G. R/R_0,_ fraction of the increase of the ratio over the baseline ratio divided by baseline ratio. **P < .01 and ***P < .001 vs EGFP, #*P* < .05 and ##*P* < .01 vs hTRPV4 or rTRPV4 (n *≥* 120 cells recorded/condition). Two-tailed *t* test for *D* and *E*, 1-way analysis of variance with Tukey’s post hoc test for *H* and *I*. The error bars indicate the standard error of the mean.

TRPV1(K710) enables direct LPA activation.^23^ Alignment of TRPV1-TRPV4 (Figure 3A) identified rat/human TRPV4(R746) and *Xenopus* TRPV4(R742) (TRPV4 structure xenopus-based^26^) as aligning with rTRPV1(K710), all within the highly conserved TRP helix. We studied relevance of TRPV4(R746) for TRPV4 activation with affirmative results, using R746D charge reversal, R746G inert function mutation, and a human arginine→cysteine polymorphism R746C (https://www.ncbi.nlm.nih.gov/clinvar/variation/VCV000450199.2) (Figure 3B–D, Supplementary Figure 6). All 3 R746 mutations activated normally with GSK101 (Supplementary Figures 6C and E, and 7). Using biochemical assays, we also demonstrated LPC(18:1)-rTRPV4 binding and observed a significant reduction of this interaction by R746D (Figure 3E). Taken together, these results strongly support that mammalian R746, located C-terminally in the TRP helix, is required for TRPV4 activation by LPC.

We used this information to build a structure-based model of LPC(18:1)/xenopus-TRPV4 binding (Figure 3F and G, Supplementary Movie 1). Considering this structural prediction, we conducted glycine mutagenesis of positively charged residues K754, R757, R774, and W776 of rTRPV4 and recorded results suggesting relevance of each residue for TRPV4 activation by LPC(18:1) (Figure 3H and I). These results might indicate a direct interaction of LPC(18:1)TRPV4, as predicted. An alternative explanation is the key relevance of these sites for activation of TRPV4 by LPC(18:1), yet binding elsewhere. Confirmation of our predicted binding site awaits future cryo-electron microscopy ultrastructural studies. Importantly, we found that the mutagenized sites did not affect GSK101 activating TRPV4 (Figure 3H and I), which likely functions via a different binding site (Supplementary Figure 8).

### TRPV4 Activation by Lysophosphatidylcholine Induces Extracellular Signal-Regulated Kinase Phosphorylation, Then Triggers Extracellular Release of MicroRNA-146a Through Rab5/ Rab27a in Skin Keratinocytes

**Figure 4.**
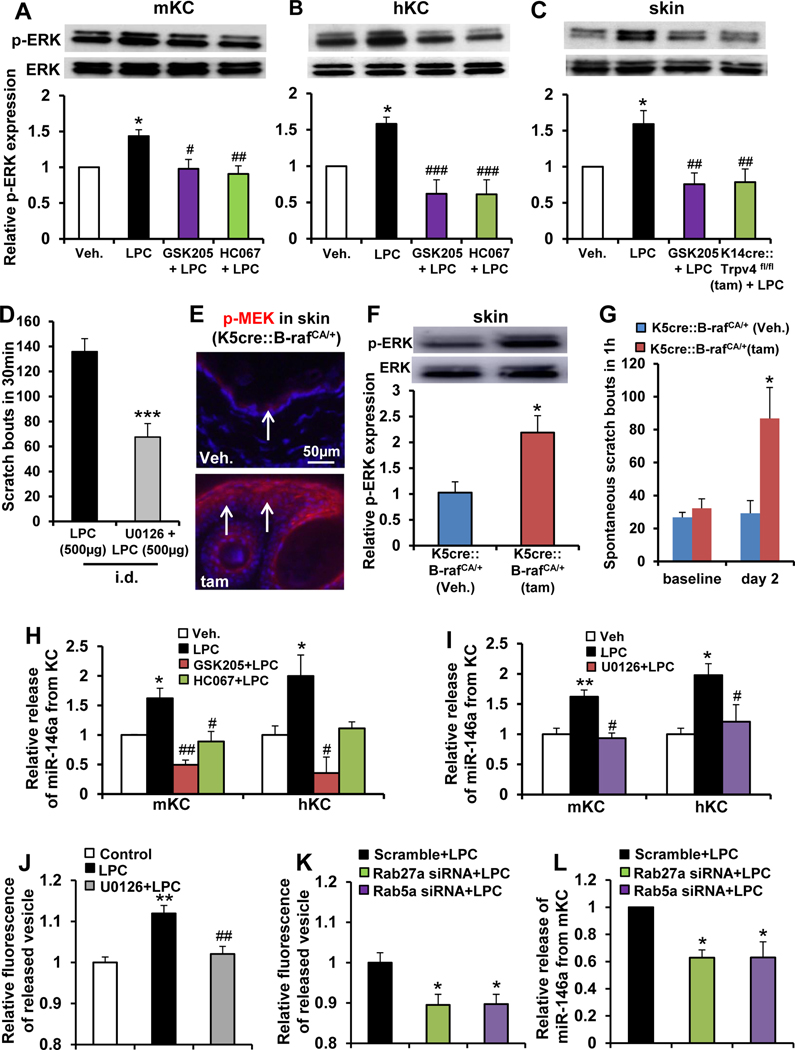
LPC elicits extracellular release of miR-146a from skin keratinocytes (KCs) depending on TRPV4→p-ERK→Rab5a/Rab27a signaling. Note LPC-induced increase of p-ERK in (*A*) cultured mouse KCs (mKCs) and (*B*) human KCs (hKCs) and its elimination by pretreatment with TRPV4 inhibitors GSK205 and HC067047 (10 *μ*mol/L) *P < .05 vs vehicle (Veh; 0.2% dimethyl sulfoxide); #*P* < .05 and ##*P* < .01 vs LPC (n = 4–6 cultures/group; 5–7 pups/culture for mice, 2 patients/culture for humans). (*C*) Intradermal (i.d.) LPC injection (500 *μ*g/50 μL) increased p-ERK in dissected dorsal neck skin that was reversed by intraperitoneal (IP) pretreatment with GSK205 (20 mg/kg) and in keratinocyte-*Trpv4* cKO mice. *P < .05 vs vehicle (Veh., normal saline), ##*P* < .01 vs LPC (n = 7 mice/group). (*D*) LPC-induced scratching was significantly attenuated by i.d. pretreatment with MEK-selective inhibitor U0126 (20 *μ*g/50 *μ*L). ***P < .001 vs LPC (n = 11 mice for LPC, n = 6 for U0126 ^+^ LPC). (*E*) Note increased p-MEK expression in dorsal neck skin 2 days after induction of the B-raf transgene by 4-hydroxy tamoxifen treatment (*arrows*: epidermis; *blue*: 4′,6-diamidino-2-phenylindole). (*F*) Western blot revealed increased p-ERK in dorsal neck skin 2 days after induction of the B-raf transgene. **P < .01 vs vehicle (n = 4 mice for vehicle and n = 7 for tamoxifen). (*G*) Induction of B-raf transgene in skin KC elicited robust scratching on day 2. *P < .05 vs vehicle (n = 5 mice for vehicle and n = 8 for tamoxifen). (*H*) LPC-induced extracellular release of miR-146a from cultured mouse and human KCs was eliminated by pretreatment with GSK205 or HC067047 (10 μmol/L). *P < .05 vs vehicle, #*P* < .05 and ##*P* < .01 vs LPC (n = 3–4 cultures/ treatment; 5–7 pups/culture for mice, 2 patients/culture for humans). (*I*) LPC-induced extracellular release of miR-146a from cultured mouse and human KCs was eliminated by pretreatment with U0126 (10 μmol/L). *P < .05 vs vehicle, #*P* < .05 and ##*P* < .01 vs LPC (n = 3–4 cultures/treatment; 5–7 pups/culture for mice, 2 patients/culture for human). (*J*) LPC-induced extracellular vesicular release from cultured mouse KC was eliminated by U0126 (10 *μ*mol/L). **P < .01 vs control, ##*P* < .01 U0126 +LPC vs LPC (n = 4–6 cultures, 5–7 pups/culture). (*K*) Experimental setup as in *J*, we detected a significant decrease of LPC-induced vesicular release from mouse KCs treated with Rab27a- small interfering (si)RNA or Rab5a-siRNA (scrambled siRNA control set ‘1’ for relative comparison). *P < .05 vs scramble +LPC (n = 4–6 cultures, 57 pups/culture). (*L*) Real-time quantitative polymerase chain reaction assay detected a significant decrease of LPC-induced release of miR-146a from mouse KCs by siRNA-mediated knockdown of Rab27a or Rab5a. *P < .05 vs scramble +LPC (n = 4–5 cultures, 5–7 pups/ culture). One-way analysis of variance with Tukey’s post hoc test was used for *A–C*, *G*, *H–L*, and 2-tailed *t* test for *D* and *F*. The *error bars* show the standard error of the mean.

We next focused on downstream signaling of TRPV4 activation by LPC to determine the mechanism(s) by which activated keratinocytes relay the signal to skin-innervating pruriceptive afferents. We focused on mitogen-activated protein kinase (MAPK) signaling based on our previous observations that mitogen-activated protein kinase (MEK)/extracellular signal-regulated kinase (ERK) activation can function downstream of TRPV4-mediated Ca^2+^ influx in keratinocytes in response to pruritogens.^10^ We detected rapidly increased ERK phosphorylation (p-ERK) in mouse and human keratinocytes 10 minutes after LPC stimulation (Figure 4A and B). This increase was TRPV4 dependent, based on effects of TRPV4 inhibitors on primary keratinocytes, and in vivo in TRPV4 inhibitor-treated mice and keratinocyte-*Trpv4* cKO mice (Figure 4C). Thus, TRPV4 is required for p-ERK increase induced by LPC in keratinocytes. Pretreatment with MEK inhibitor U0126 (ID) caused a significant reduction in LPC-induced scratching (Figure 4D). Mice with a constitutively active B-raf transgene were used to address whether activation of MAP kinases in skin keratinocytes suffices to elicit itch. Upon transgene induction, we observed significantly increased pMEK/p-ERK in skin and robust spontaneous scratching (Figure 4E–G). Thus, keratinocyte p-ERK is necessary and sufficient for LPC-induced itch.

We then queried paracrine-secretory functions of keratinocytes that underlie activation of skin-innervating pruriceptor nerve endings. We focused on secreted miRs because miRs can signal paracrine directly via cell surface receptors such as Toll-like receptors (TLRs) or TRP channels (eg, TRPA1), which have been found expressed by peripheral sensory neurons and involved in itch and pain.^27,28^ We decided to test miR-let-7b, miR-125b-1, miR-16–5p, miR203, and miR-146a because they are abundantly expressed in skin and involved in skin inflammation.^29,30^ We observed LPC evoked an increased release of miR146a from mouse and human keratinocytes (Figure 4H and I) but had no effect on other miRs (Supplementary Figure 9). In addition, miR146a release was TRPV4 and MEK/ERK dependent (Figure 4H and I). Our results agree with previous findings that MEK/ERK inhibition suppresses vesicular biogenesis and secretion.^31^ We then generated additional evidence for LPC-induced vesicular release and extracellular release of miR-146 by knocking down Rab5a and Rab27a, both known components of the cellular vesicular release machinery and downstream targets of MEK/ERK in vesicular release,^31–33^ which significantly attenuated both processes (Figure 4J–L)

### MicroRNA-146a is a Pruritogen and Functions via TRPV1 in Primary Pruriceptor Neurons

**Figure 5.**
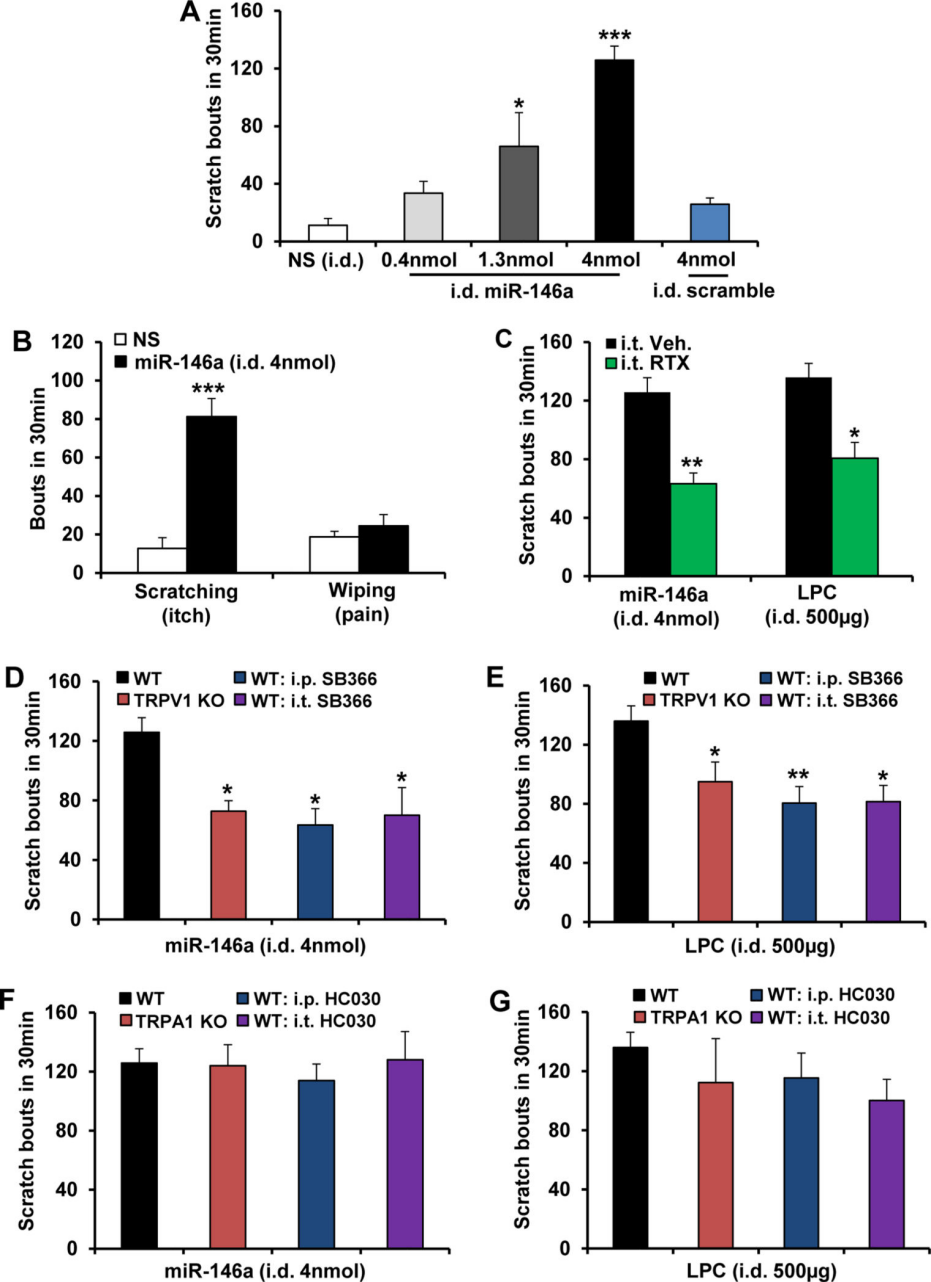
miR-146a elicits scratching behavior, which requires TRPV1, but not TRPA1, in sensory neurons. (*A*) miR-146a induced dose-dependent scratching behavior, but scramble control did not cause significant scratching behavior. i.d., intradermal. *P < .05 and ***P < .001 vs normal saline (NS) (n = 4–5 mice/group). (*B*) The mouse cheek model demonstrated that miR-146a elicited robust scratching (itch) but not wiping response (pain). ***P < .001 vs NS (n = 4–5 mice/group). (*C*) Elimination of TRPV1-expressing spinal nerve terminals using intrathecal (i.t.) injection of resiniferatoxin (RTX, 200 ng/5 *μ*L) significantly reduced miR-146a– or LPC-induced itch. *P < .05 and **P < .01 vs vehicle (Veh, 2% ethanol ^+^ 2% Tween 80) (n = 4–5 mice/group except n = 11 for vehicle ^+^ LPC). (*D* and *E*) miR-146a– and LPC-induced itch were significantly attenuated by intraperitoneal (i.p.) (2 mg/kg) or i.t. (30 *μ*g) injection of the TRPV1 inhibitor SB366791, or in Trpv1 KO. *P < .05 and **P < .01 vs WT (n = 4–5 mice/group for D and n = 6–11 mice/group for E). Itch induced by (*F*) miR-146a (n = 4–5 mice/group) and (*G*) LPC (n = 5–11 mice/group) were not significantly altered by i.p. (30 mg/kg) or i.t. (30 *μ*g) injection of the TRPA1-inhibitor HC030031, or in *Trpa1* KO. One-way analysis of variance with Tukey’s post hoc test was used for *A* and *D–G*, 2-tailed *t* test for *B* and *C*. The *error bars* show the standard error of the mean.

To address whether miR-146a is pruritic, we ID injected mice and observed dose-dependent scratching (Figure 5A), not pain-related wiping (Figure 5B). This scratching response to miR-146a (4 nmol/50 *μ*L) was immediate (latencies: 25 seconds for miR-146a vs 55 seconds for LPC), indicating nondelayed signaling of miR-146a to skin-innervating peripheral pruriceptor axons as opposed to an indirect mechanism, such as gene regulation, as known for microRNAs. We next tested whether miR-146a–induced itch relied on TRPV1^+^ pruriceptor neurons, given the established role of TRPV1 in these neurons.^9^ Selective ablation of TRPV1^+^ central nerve terminals by IT injection of resiniferatoxin (Supplementary Figure 10) resulted in significantly reduced miR-146a–induced scratching, similar for LPC (Figure 5C). Thus the signaling chain LPC→keratinocyte-TRPV4→extracellularly released miR-146a relies on TRPV1^+^ pruriceptor neurons for causing itch. These results were extended by observing that itch induced by miR146a– or LPC-induced itch was significantly reduced by KO or inhibition of TRPV1, but not TRPA1, indicating a reliance of pruritic effects of miR-146a and LPC on TRPV1 (Figure 5D–G). Notably, however, none of our TRPV1targeting approaches completely eliminated scratching, suggesting additional contributory signaling mechanisms.

**Figure 6.**
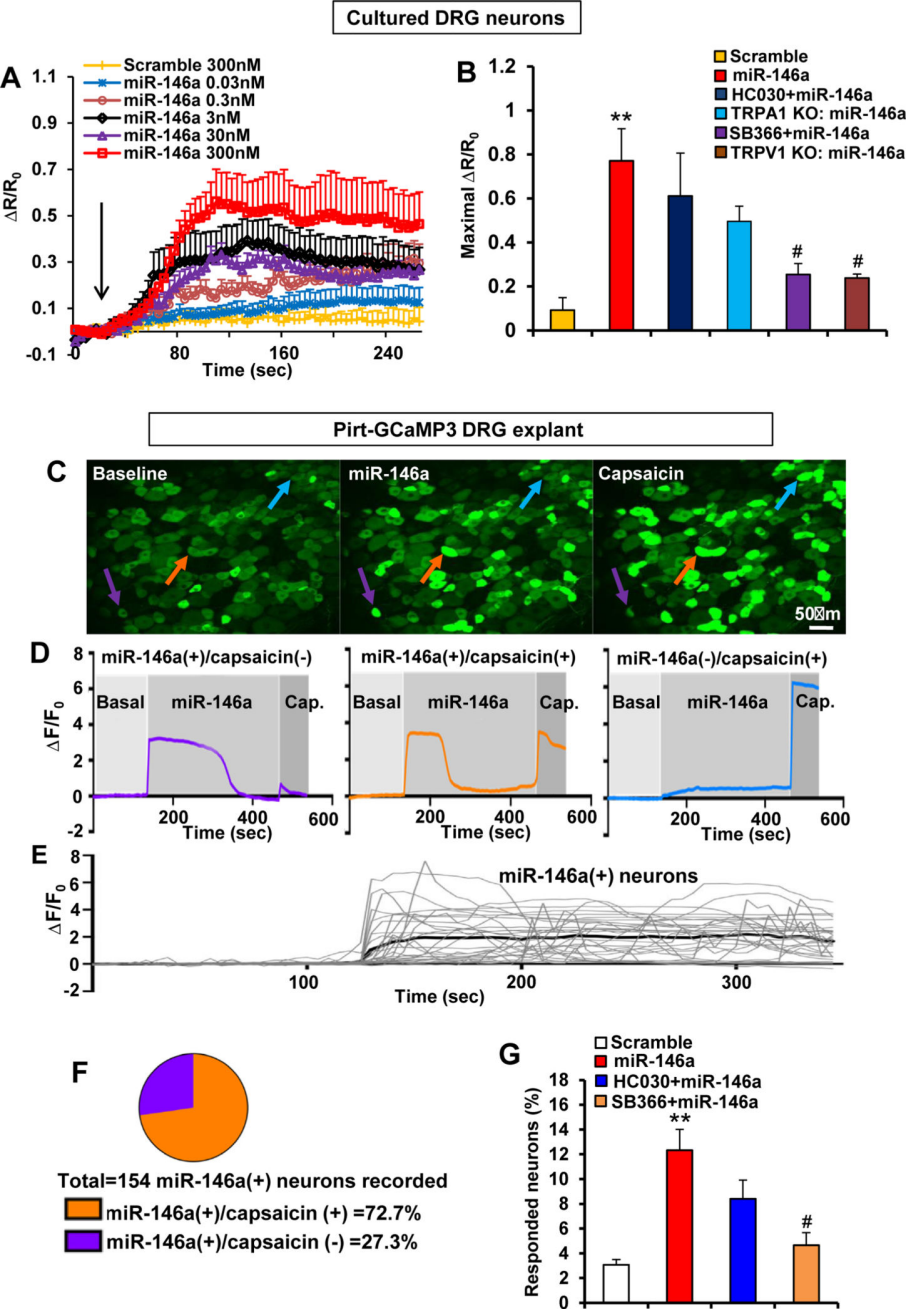
miR-146a activates primary sensory neurons in a TRPV1- but not TRPA1-dependent manner. (*A*) miR-146a induced Ca^2+^ influx in cultured DRG neurons (n ≥ 190 neurons recorded/concentration) in a dose-dependent manner (*arrow*: miR-146a or scramble stimulation). R/R_0_ is the fraction of the increase of a given ratio over baseline ratio divided by baseline ratio. (*B*) miR-46a (300 nmol/L) induced Ca^2+^ influx that was significantly reduced by pretreatment with TRPV1 inhibitor SB366791 (10 *μ*mol/L) and in neurons (n ≥ 190 neurons recorded/concentration) from *Trpv1* KO mice but was not significantly altered by TRPA1 inhibitor HC030031 (10 *μ*mol/L) or in neurons from *Trpa1* KO mice. R/R_0_ is the fraction of the increase of the ratio over the baseline ratio divided by baseline ratio **P < .01 vs scramble (300 nmol/L) and #*P* < .05 vs miR-146a. (*C*) Representative Ca^2+^ imaging of GCaMP3-expressing DRG neurons in an ex vivo preparation illustrates the increased Ca^2+^ signal (arrows; colors matching the Ca^2+^ transients in D) after stimulation with miR-146a (300 nmol/L) and capsaicin (1 *μ*mol/L). (*D*) Representative Ca^2+^ traces of miR-146a(+)/capsaicin(−), miR-146a(+)/capsaicin(−), or miR-146a(−)/capsaicin(+) DRG neurons. F/ F_0_ is the ratio of fluorescence difference to baseline. (*E*) Representative Ca^2+^ traces of a population of DRG neurons responsive to miR-146a. (*F*) Of 1250 neurons recorded, 154 were responsive to miR-146a, and 72.7% of miR-146a–responsive neurons were also capsaicin responsive. (*G*) Increased percentage of total DRG neurons responding to miR-146a (300 nmol/L) was significantly reduced by SB366791 (10 mmol/L) but not by HC030031 (10 mmol/L). **P < .01 vs scramble-control (300 nmol/L) and #*P* < .05 vs miR-146a (n = 4–9 DRG explants/group (1 explant /mouse). One-way analysis of variance with Tukey’s post hoc test was used for *B* and *G*. The error bars indicate the standard error of the mean.

To elucidate sensory neuronal signaling in response to miR-146a, we recorded miR-146a–induced Ca^2+^ influx in a dose-dependent manner in dissociated DRG neurons (Figure 6A). Ca^2+^ influx depended on TRPV1 but not TRPA1, as observed when using selective inhibitors and *Trpv1* KO and Trpa1 KO dissociated neurons (Figure 6B). We extended these findings to an organotypic DRG preparation, which enables Ca^2+^ imaging via *Pirt* promoter-driven Ca^2+^ indicator GCaMP3. miR-146a evoked a neuronal Ca^2+^ transient in DRGs (Figure 6C–E, Supplementary Movie 2). Responsive neurons were ∼12% of DRG neurons (Figure 6G), and 72.7% of those were capsaicin responsive (Figure 6F). The percentage of miR-146a–responding neurons was significantly reduced when inhibiting TRPV1 but not TRPA1 (Figure 6G).

These findings support the novel concept that extracellularly released miR-146a from skin keratinocytes in response to LPC-induced TRPV4 activation activates skin-innervating TRPV1^+^ pruriceptor neurons. Their peripheral epidermal projections are activated in a paracrine manner to elicit itch. Recent studies have demonstrated that extracellular miRs can directly or indirectly via TLR7 activate TRPA1 to elicit pain or itch^27,28^ and that activation of TLR2/TLR6 heterodimers induces pain through TRPV1 and TRPA1.^34^ Here, we could not corroborate that miR-146a– induced itch was significantly influenced by KO or inhibition of TLR7 or inhibition of TLR2/6. In addition, we did not see significant Ca^2+^ influx upon stimulation with miR-146a in HEK293 cells transfected with rTRPV1 or cotransfected with rTRPV1 and rTLR7, rTLR2, or rTLR6 (Supplementary Figure 11).

Pruritic neural signaling can be attenuated by activation of *k*-opioid receptors, which has gained in clinical relevance with antipruritic effects.^35^ We recorded no effect of k-opioid receptor agonism on LPC and miR-146a pruritogenicity, indicating their mutual independence (Supplementary Figure 12).

Future studies will elucidate the mechanisms of how miR-146a activates TRPV1^+^ pruriceptor neurons at increased resolution.

### Lysophosphatidylcholine and MicroRNA-146a Are Elevated in Cholestatic Pruritic Mice and in Patients With Cholestatic Itch

**Figure 7.**
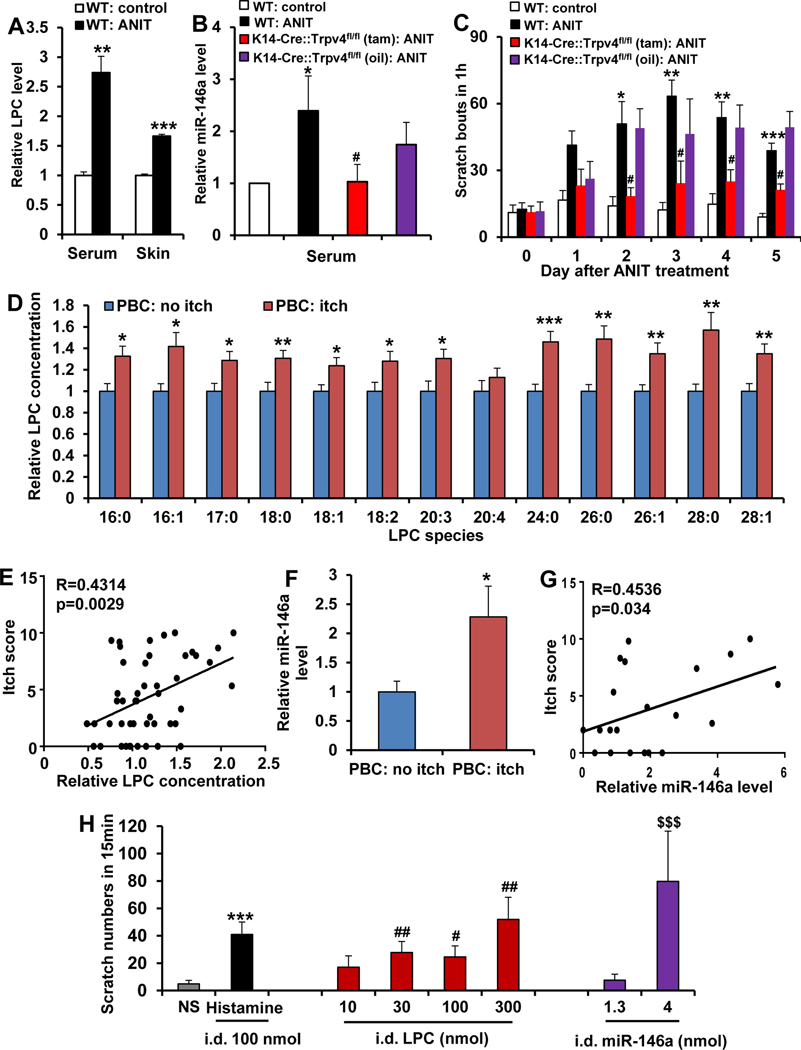
LPC and miR-146a are elevated in mice or patients with PBC with cholestatic itch and induced itch in nonhuman primates. (*A*) ANIT treatment increased LPC levels in serum and skin. **P < .01 and ***P < .001 vs WT-control, 2-tailed *t* test (n = 5–8 mice/group). (*B*) ANIT treatment increased miR-146a in serum, which was attenuated in keratinocyte-*Trpv4* cKO mice. *P < .05 vs WT control, and #*P* < .05 vs keratinocyte-*Trpv4* cKO mice, 1-way analysis of variance with Tukey’s post hoc test (n = 8–11 mice/group). (*C*) ANIT-induced cholestatic itch was significantly attenuated in keratinocyte-Trpv4 cKO mice. *P < .05, **P < .01, ***P < .001 vs WT control; #*P* < .05 vs #*P* < .05 vs keratinocyte-Trpv4 cKO mice, 2-way analysis of variance with Tukey’s post hoc test (n = 6 mice/group). (*D*) All detected LPC species, except for LPC(20:4), were significantly elevated in sera of patients with PBC with itch (n = 27) vs without itch (n = 21), *P < .05 and **P < .01, 2-tailed t test. (*E*) When all detected LPC species from individual patients were aggregated, there was a significant correlation of total LPC concentration with the itch intensity. Pearson’s correlation coefficient, *R* = 0.4314; *P* =.0029. (*F*) A significant increase in abundance of miR-146a was detected in sera of patients with PBC with itch (n = 10) vs without itch (n = 12), **P < .01, 2-tailed t test. (*G*) Note a significant correlation of miR-146a level with the itch intensity. Pearson’s correlation coefficient, *R* = 0.4536; *P* = .034. (*H*) LPC or miR146a induced scratching behavior in rhesus monkeys in a dose-dependent manner. Histamine was used as positive control. i.d., intradermal. ***P < .001, #*P* < .05, ##*P* < .01, and $ $ $P < .001 vs normal saline (NS), repeated measures using a linear mixed model (n = 9 monkeys/group). The *error bars* indicate the standard error of the mean.

Next we addressed the role of keratinocyte-TRPV4 in cholestatic itch and whether LPC and miR-146a function as pruritogens in a cholestasis disease-relevant context. We measured LPC and miR-146a levels in a mouse cholestasis model induced by systemic injection of ANIT.^5^ We detected significantly increased LPC in both sera and skin (Figure 7A) and elevated systemic miR-146a in ANIT-treated mice (Figure 7B). ANIT-induced cholestatic itch (Figure 7C) was almost eliminated in keratinocyte-Trpv4 cKO mice. This indicates a key role for keratinocytes and keratinocyte-*TRPV4* signaling in cholestatic itch. Moreover, systemic miR-146a was significantly reduced in keratinocyte-*Trpv4* cKO ANIT mice (Figure 7B), indicating keratinocytes’ release of miR146a depended on cell-autonomous TRPV4 signaling.

We next measured LPC concentrations in sera of patients with PBC, an immune-mediated cholestatic liver disease with a high prevalence of pruritus, vs control patients with normal liver biopsy specimens, observing no significant differences (Supplementary Figure 13). However, itchphenotypic information was not available for these patients (Duke Health System).

We therefore tapped into patient biobank repositories where itch phenotypic information was readily available (Warsaw Medical University and University Hospital of Erlangen). We detected a significant increase of the vast majority of LPC species in sera of patients with PBC with itch vs no itch (Figure 7D). Itch intensity correlated with LPC concentrations (*R* = 0.4314, *P* < .0029; Figure 7E). We also found miR-146a levels were significantly elevated and correlated with itch intensity (Figure 7F and G). These findings, together with our other data, support the concept that upregulation of systemic LPC and miR-146a contributes to pruritus in PBC.

### Lysophosphatidylcholine and MicroRNA-146a Evoke Itch in Nonhuman Primates

We next addressed whether LPC and miR-146a are pruritogens in primates. Both molecules, upon ID injection, induced pruritus in rhesus monkeys in a dose-dependent manner (Figure 7H). This suggests that our new pruritus mechanism in mice and human primary keratinocytes extends to primates, further supporting our premise that LPC and miR-146a contribute to cholestatic itch.

## Discussion

Here we describe a new signaling pathway of skin→sensory neuron cross talk relevant for cholestatic itch (Supplementary Figure 14). Our findings characterize the skin as a hitherto overlooked critical participant in cholestatic itch. Cholestatic itch has been viewed as the diseased liver generating pruritogenic mediators, which then sensitize and activate pruriceptor sensory neurons to evoke perception of itch via neural transmission.^3,5,8^ Remarkably, this paradigm has not offered much about (1) why the itch sensation originates from the epidermis, (2) molecular mechanisms in skin cells, and (3) how skin cells communicate with pruriceptor neurons’ peripheral axons in cholestatic itch.

Our findings provide an initial answer, and we deconstruct “forefront” signaling involving epidermal keratinocytes responding to LPC via TRPV4 activation, then signaling by extracellular release of miR-146a to TRPV1^+^ skin-innervating peripheral nerve endings, activating these pruriceptors. We identified as novel pruritogen LPC, which is biosynthetically upstream of LPA, a previously implicated pruritogen in cholestatic itch. Elevated LPC and miR-146a were detected systemically in patients with PBC and mice with cholestatic itch. LPC and miR-146a were pruritic in mice and nonhuman primates. We observed that the pruritogenic effects of LPC and cholestatic itch in mice relied on keratinocyte-TRPV4. In addition, direct activation of TRPV4 by LPC led to Ca^2+^ influx into keratinocytes, which triggered MEK-ERK MAP kinase signaling, which in turn evoked extracellular release of miR-146a relying on Rab5a and Rab27a, known components of the cellular vesicular release machinery.

Translational medical relevance rests on the following: Because LPC and miR-146a were elevated in the blood of patients with PBC, the concept of LPC and miR-146a as possible biomarkers of cholestatic itch can now be addressed. Possibly our findings also apply to other hepatic pruritic diseases, another interesting subject for future studies. We note that elevated LPC was previously observed in patients with uremic pruritus,^14^ psoriasis,^11,12^ and atopic dermatitis.^13^

Back to cholestatic itch, this condition is almost certainly not monofactorial. Most likely other metabolites function in a co-contributory manner, namely, bilirubin, bile acids, and LPA.^2,3,5,6^ However, it is clear that (1) LPC is a robust pruritogen, (2) LPC has its own unique pathway in itch, yet conversion of LPC into LPA contributes to LPC pruritogenicity, (3) systemic LPC concentrations are significantly elevated in patients with PBC and pruritus and correlated with itch intensity, and (4) systemic and skin LPC concentrations are significantly elevated in cholestatic mice.

Furthermore pertinent to translation, several molecules in the readily targetable integument can be targets, including TRPV4, TRPV1, MEK/ERK, and miR-146a. We also believe that our results of pruritogenicity of LPC and miR146a in primates are relevant. Of note, a previous study in human volunteers demonstrated that ID injection of LPC caused histopathology of skin irritation and inflammation, but pruritogenicity was not tested.^36^ Decades before, LPC-evoked localized allergic inflammation in human skin was reported.^37^

Another recent study on TRPV4 expression in human chronic pruritus appears relevant. Chronic pruritus was associated with individual increased epidermal TRPV4 expression, and these patients had increased responses to capsaicin, including pruritus and burning/warmth sensation.^38^ This finding, namely, that increased TRPV4 expression in pruritic skin sensitizes TRPV1 signaling in sensory neurons, appears in agreement with our study.

miRNAs are small, highly conserved, noncoding RNA molecules with known roles in RNA silencing and posttranscriptional regulation of gene expression.^39^ Interestingly, recent work discovered an unconventional role for miRs: they can either directly or indirectly activate TRPA1 in sensory neurons to induce itch or pain.^27,28^ miR-146a has been found immunomodulatory in inflammation with postulated proresolution roles in psoriasis and atopic dermatitis, both of which are pruritic.^29,30,40^

Our experiments support a new aspect of miR-146a in skin and sensory biology, namely, that extracellular release of miR-146a from keratinocytes acts as a “transmitter” between keratinocytes and skin-innervating sensory neurons to primarily trigger itch. Indeed, ID injection of miR-146a elicited a more rapid scratching response (latency: ∼25 seconds) than that of LPC (∼55 seconds), suggesting that (1) miR-146a induces itch without affecting gene regulation and that (2) it functions downstream of LPC in itch. In addition, miR-146a did not elicit pain behavior in the mouse cheek model, indicating its selective activation of neural itch pathways. We reiterate that the mechanism of how miR146a activates TRPV1^+^ sensory neurons needs to be further elucidated, especially miR-146a evoking itch, not pain.

Our findings are basic science relevant. We implicated a putative new binding site for LPC(18:1) in the C-terminus of TRPV4, directly adjacent to the TRP helix. The widely used synthetic activator GSK101 does not bind here and activates heterologously expressed TRPV4 with higher single-channel conductance than the natural activator, LPC(18:1). Indeed, our discovery defines the first endogenous glycerophospholipid activator of TRPV4. Given that GSK101 is lethal in vivo,^41^ the quest for a therapeutically beneficial (eg, in arthritis, hepatic, or renal disease) TRPV4 activator continues. Our discovery of a putative LPC(18:1)-TRPV4 binding site, awaiting confirmation in future structural studies, provides a framework for development of nonlethal TRPV4activating molecules and inverse agonists. Also, TRPV4^0^s gating mechanism can now be interrogated by comparing LPC(18:1) bound to TRPV4 vs GSK101 bound to TRPV4 using structural methods.

We view our discovery as a novel concept in the sensory submodality of cholestatic itch. The signaling mechanism we have unveiled depends on the following critical players: the innervated epithelia and the innervating pruriceptor sensory neuron, both of which rely on unexpected signaling molecules. We identified a hitherto nonrecognized glycerophospholipid, LPC, as a pruritogen that initiates the signaling cascade in the skin, plus the messenger of the epithelia-sensory neuron cross talk, an immunomodulatory microRNA, miR-146a, that directly activates pruriceptor sensory neurons. TRPV4 on keratinocytes and TRPV1 on pruriceptor sensory neurons function synergistically as key molecular players in this debilitating form of itch.

## Supplementary Material

Video S1

Video S2

1

## WHAT YOU NEED TO KNOW SUMMARY

### BACKGROUND AND CONTEXT

Mechanistic understanding of cholestatic pruritus is lacking, for example, in primary biliary cholangitis, an autoimmune hepatopathy.

### NEW FINDINGS

Lysophosphatidylcholine (LPC) functions as a novel cholestatic pruritogen. As a mechanism, LPC directly activates TRPV4 ion channels of skin keratinocytes, which triggers the release of microRNA-146a to activate TRPV1^+^ pruriceptor neurons.

### LIMITATIONS

How microRNA-146a activates TRPV1^+^ pruriceptor neurons needs to be further elucidated.

### IMPACT

LPC functions as pruritogen with potential impact in primary biliary cholangitis. This new LPC–TRPV4-based mechanism supports the novel concept of the skin as a forefront sensory organ, relevant in cholestatic itch.

## Abbreviations used in this paper:

ANIT: *α*-naphthyl-isothiocyanate
DRG: dorsal root ganglion
ERK: extracellular signal-regulated kinase
ID: intradermal
IT: intrathecal
KO: knockout
LPA: lysophosphatidic acid
LPC: lysophosphatidylcholine
MEK: mitogen-activated protein kinase
miR: microRNA
PBC: primary biliary cholangitis
TRP: transient receptor potential
HEK: human embryonic kidney
h: human
r: rat
p-: phosphorylated
tam: tamoxifen
TLR: Toll-like receptors
WT: wild-type

## References

[1] Dull MM, Kremer AE. Treatment of pruritus secondary to liver disease. Curr Gastroenterol Rep 2019;21:48. 31367993 10.1007/s11894-019-0713-6

[2] Kremer AE, Martens JJ, Kulik W, Lysophosphatidic acid is a potential mediator of cholestatic pruritus. Gastroenterology 2010;139:1008–1018. 20546739 10.1053/j.gastro.2010.05.009

[3] Lieu T, Jayaweera G, Zhao P, The bile acid receptor TGR5 activates the TRPA1 channel to induce itch in mice. Gastroenterology 2014;147:1417–1428. 25194674 10.1053/j.gastro.2014.08.042 PMC4821165

[4] Abu-Hayyeh S, Ovadia C, Lieu T, Prognostic and mechanistic potential of progesterone sulfates in intrahepatic cholestasis of pregnancy and pruritus gravidarum. Hepatology 2016;63:1287–1298. 26426865 10.1002/hep.28265 PMC4869673

[5] Meixiong J, Vasavda C, Snyder SH, MRGPRX4 is a G protein-coupled receptor activated by bile acids that may contribute to cholestatic pruritus. Proc Natl Acad Sci U S A 2019;116:10525–10530. 10.1073/pnas.1903316116 PMC6535009 31068464

[6] Yu H, Zhao T, Liu S, MRGPRX4 is a bile acid receptor for human cholestatic itch. Elife 2019;8:e48431. 10.7554/eLife.48431 PMC6773440 31500698

[7] Noguchi K, Herr D, Mutoh T, Lysophosphatidic acid (LPA) and its receptors. Curr Opin Pharmacol 2009; 9:15–23. 19119080 10.1016/j.coph.2008.11.010

[8] Kittaka H, Uchida K, Fukuta N, Lysophosphatidic acid-induced itch is mediated by signalling of LPA5 receptor, phospholipase D and TRPA1/TRPV1. J Physiol 2017;595:2681–2698. 28176353 10.1113/JP273961 PMC5390871

[9] Moore C, Gupta R, Jordt SE, Regulation of pain and itch by TRP channels. Neurosci Bull 2018;34:120–142. 29282613 10.1007/s12264-017-0200-8 PMC5799130

[10] Chen Y, Fang Q, Wang Z, Transient receptor potential vanilloid 4 ion channel functions as a pruriceptor in epidermal keratinocytes to evoke histaminergic itch. J Biol Chem 2016;291:10252–10262. 10.1074/jbc.M116.716464 PMC4858974 26961876

[11] Zeng C, Wen B, Hou G, Lipidomics profiling reveals the role of glycerophospholipid metabolism in psoriasis. Gigascience 2017;6:1–11. 10.1093/gigascience/gix087 PMC5647792 29046044

[12] Ryborg AK, Gron B, Kragballe K. Increased lysophosphatidylcholine content in lesional psoriatic skin. Br J Dermatol 1995;133:398–402. 8546994 10.1111/j.1365-2133.1995.tb02667.x

[13] Berdyshev E, Goleva E, Bronova I, Lipid abnormalities in atopic skin are driven by type 2 cytokines. JCI Insight 2018;3:e98006. 10.1172/jci.insight.98006 PMC5916244 29467325

[14] Wu Q, Zhang H, Ding JR, UPLC-QTOF MS-based serum metabolomic profiling analysis reveals the molecular perturbations underlying uremic pruritus. Biomed Res Int 2018;2018:4351674. 10.1155/2018/4351674 PMC5818897 29546058

[15] Kim YS, Chu Y, Han L, . Central terminal sensitization of TRPV1 by descending serotonergic facilitation modulates chronic pain. Neuron 2014; 81:873–887. 24462040 10.1016/j.neuron.2013.12.011 PMC3943838

[16] Lee H, Ko MC. Distinct functions of opioid-related peptides and gastrin-releasing peptide in regulating itch and pain in the spinal cord of primates. Sci Rep 2015;5:11676. 10.1038/srep11676 PMC4483774 26119696

[17] Hamill OP, Marty A, Neher E, Improved patch-clamp techniques for high-resolution current recording from cells and cell-free membrane patches. Pflugers Arch 1981;391:85–100. 6270629 10.1007/BF00656997

[18] Morales-Lázaro SL, Llorente I, Sierra-Ramírez F, Inhibition of TRPV1 channels by a naturally occurring omega-9 fatty acid reduces pain and itch. Nat Commun 2016;7:13092. 10.1038/ncomms13092 PMC5062500 27721373

[19] Malik ZA, Liu TT, Knowlton AA. Cardiac myocyte exosome isolation. Methods Mol Biol 2016;1448:237–248. 27317185 10.1007/978-1-4939-3753-0_17 PMC5693424

[20] Kishimoto T, Soda Y, Matsuyama Y, An enzymatic assay for lysophosphatidylcholine concentration in human serum and plasma. Clin Biochem 2002;35:411–416. 12270773 10.1016/s0009-9120(02)00327-2

[21] Kittaka H, Tominaga M. The molecular and cellular mechanisms of itch and the involvement of TRP channels in the peripheral sensory nervous system and skin. Allergol Int 2017;66:22–30. 28012781 10.1016/j.alit.2016.10.003

[22] Moehring F, Mikesell AR, Sadler KE, Piezo1 mediates keratinocyte mechanotransduction. bioRxiv 2020. 2020.07.19.211086.

[23] Nieto-Posadas A, Picazo-Juarez G, Llorente I, Lysophosphatidic acid directly activates TRPV1 through a C-terminal binding site. Nat Chem Biol 2011;8:78–85. 22101604 10.1038/nchembio.712

[24] Takahashi N, Hamada-Nakahara S, Itoh Y, TRPV4 channel activity is modulated by direct interaction of the ankyrin domain to PI(4,5)P(2). Nat Commun 2014;5:4994. 25256292 10.1038/ncomms5994

[25] Garcia-Elias A, Mrkonjic S, Pardo-Pastor C, Phosphatidylinositol-4,5–biphosphate-dependent rearrangement of TRPV4 cytosolic tails enables channel activation by physiological stimuli. Proc Natl Acad Sci U S A 2013;110:9553–9558. 23690576 10.1073/pnas.1220231110 PMC3677448

[26] Deng Z, Paknejad N, Maksaev G, Cryo-EM and Xray structures of TRPV4 reveal insight into ion permeation and gating mechanisms. Nat Struct Mol Biol 2018; 25:252–260. 29483651 10.1038/s41594-018-0037-5 PMC6252174

[27] Park CK, Xu ZZ, Berta T, Extracellular microRNAs activate nociceptor neurons to elicit pain via TLR7 and TRPA1. Neuron 2014;82:47–54. 24698267 10.1016/j.neuron.2014.02.011 PMC3982230

[28] Han Q, Liu D, Convertino M, miRNA-711 Binds and activates TRPA1 extracellularly to evoke acute and chronic pruritus. Neuron 2018;99:449–463.e6. 10.1016/j.neuron.2018.06.039 PMC6091677 30033153

[29] Sonkoly E, Stahle M, Pivarcsi A. MicroRNAs: novel regulators in skin inflammation. Clin Exp Dermatol 2008; 33:312–315. 18419608 10.1111/j.1365-2230.2008.02804.x

[30] Banerjee J, Sen CK. MicroRNAs in skin and wound healing. Methods Mol Biol 2013;936:343–356. 23007520 10.1007/978-1-62703-083-0_26 PMC4357320

[31] Datta A, Kim H, Lal M, Manumycin A suppresses exosome biogenesis and secretion via targeted inhibition of Ras/Raf/ERK1/2 signaling and hnRNP H1 in castration-resistant prostate cancer cells. Cancer Lett 2017;408:73–81. 28844715 10.1016/j.canlet.2017.08.020 PMC5628151

[32] Colombo M, Raposo G, Théry C. Biogenesis, secretion, and intercellular interactions of exosomes and other extracellular vesicles. Annu Rev Cell Dev Biol 2014; 30:255–289. 25288114 10.1146/annurev-cellbio-101512-122326

[33] Datta A, Kim H, McGee L, High-throughput screening identified selective inhibitors of exosome biogenesis and secretion: a drug repurposing strategy for advanced cancer. Sci Rep 2018;8:8161. 29802284 10.1038/s41598-018-26411-7 PMC5970137

[34] Wang TT, Xu XY, Lin W, Activation of different heterodimers of TLR2 distinctly mediates pain and itch. Neuroscience 2020;429:245–255. 31954829 10.1016/j.neuroscience.2020.01.010

[35] Tseng PY, Hoon MA. Molecular genetics of kappa opioids in pain and itch sensations. Handb Exp Pharmacol. Published online ahead of print, Nov 4, 2020. 10.1007/164_2020_397 PMC9549967 33145633

[36] Ryborg AK, Deleuran B, Sogaard H, Intracutaneous injection of lysophosphatidylcholine induces skin inflammation and accumulation of leukocytes. Acta Derm Venereol 2000;80:242–246. 11028854 10.1080/000155500750012090

[37] Middleton E Jr, Phillips GB. Release of histamine activity in human skin by lysolecithin. J Lab Clin Med 1964; 64:889–894. 14239951

[38] Hidding J, Agelopoulos K, Pereira MP, Sensory qualities point to different structural and functional skin patterns in chronic pruritus patients. A translational explorative study. Acta Derm Venereol 2019;99:668–674. 30938826 10.2340/00015555-3188

[39] Bartel DP. MicroRNAs: genomics, biogenesis, mechanism, and function. Cell 2004;116:281–297. 14744438 10.1016/s0092-8674(04)00045-5

[40] Rebane A, Runnel T, Aab A, MicroRNA-146a alleviates chronic skin inflammation in atopic dermatitis through suppression of innate immune responses in keratinocytes. J Allergy Clin Immunol 2014;134:836–847. e11. 10.1016/j.jaci.2014.05.022 24996260

[41] Pankey EA, Zsombok A, Lasker GF, Analysis of responses to the TRPV4 agonist GSK1016790A in the pulmonary vascular bed of the intact-chest rat. Am J Physiol Heart Circ Physiol 2014;306:H33–H40. 24186096 10.1152/ajpheart.00303.2013 PMC3920159

