# Supplementary material for: Epithelia-Sensory Neuron Cross Talk Underlies Cholestatic Itch Induced by Lysophosphatidylcholine
Source: Gastroenterology. Author manuscript; Available in PMC 2022 Jul 1. (PMC9093619; doi:10.1053/j.gastro.2021.03.049)
Supplement: 1 [file NIHMS1692682-supplement-1.docx]

**Supplementary Materials and Methods**

**Animals**

Wild-type (WT) C57bl/6j mice were purchased from the Jackson Laboratory*. Trpv4* knockout (KO) mice were generated in our laboratory as previously described.^1^ *Trpv1* KO (Stock No: 003770), *Trpa1* KO (Stock No: 006401), and *Tlr7* KO (Stock No: 008380) mice, originally obtained from the Jackson Laboratory, were provided by Dr. Ru-Rong Ji at Duke University. Pirt-GCaMP3 mice,^2^ originally generated by Dr. Xinzhong Dong at Johns Hopkins University, were provided by Dr. Andrea Nackley at Duke University. Pirt-GCaMP3 mice express the calcium indicator, GCaMP3, in >96% of primary sensory neurons in the dorsal root ganglion (DRG) and trigeminal ganglion (TG).

Keratinocyte-specific, tamoxifen (tam)-inducible *Trpv4* knockout mice were used as previously described.^3-5^ In brief, the *Trpv4* genomic locus was engineered so that loxP sites surrounded exon 13, which encodes TM5–6. This mutation was propagated in mice that were crossed to K14-Cre-ER^tam^ mice, so that K14-Cre-ER^tam^::*Trpv4*^lox/lox^ mice could be induced by tamoxifen (tam) administration via oral gavage for five consecutive days at 5 mg/day in 0.25 ml corn oil at 2–2.5 months of age, plus a booster 2 weeks after the last application. Control animals received the same volume of corn oil. Efficiency of targeting was verified by quantitative real-time PCR and immunohistochemistry for *Trpv4* expression in skin at gene and protein levels, respectively.^3^

We also generated mice with deletion for *Trpv4* in primary sensory neurons via Cre-loxP-mediated recombination by mating mice carrying *Trpv4* (Trpv4^fl/fl^) with a mouse line expressing Cre recombinase under control of the *Nav1.8* promoter (Nav1.8-Cre). The Cre mice enable gene recombination commencing at birth selectively in sensory neurons expressing the sodium channel Nav1.8, without affecting gene expression in the spinal cord, brain, or any other organ in the body.^6^ Efficiency of targeting was verified by quantitative real-time PCR and immunohistochemistry for *Trpv4* expression in both DRGs and TGs at gene and protein levels, respectively (Supplementary Figure.1).

We also generated mice with inducible expression of constitutively active B-raf(V600E) in keratinocytes by crossing mice with a floxed allele for B-raf (B-raf^CA/+^ mice) ^7^ with K5-cre-ER^tam^ mice.^8^ The generated K5-cre-ER^tam^::Braf^CA/+^ mice (provided by Dr. Jennifer Zhang at Duke Uni.) were shaved at the dorsal back and topically treated with 4-hydroxy tamoxifen (100 µl of 20 mg/ml) in ethanol (EtOH) for 2 consecutive days. Control animals received the same volume of EtOH. Increased levels of p-MEK and p-ERK, downstream targets of B-raf, were detected in skin after treatment with 4-hydroxy tamoxifen, as verified by immunohistochemistry or Western blot (Figure. 4E-F).

Mice were housed in climate-controlled rooms on a 12/12-h light/dark cycle with water and a standardized rodent diet available *ad libitum*. All animal protocols were approved by the Duke University Institutional Animal Care and Use Committee (IACUC) in compliance with National Institutes of Health (NIH) guidelines. All of these mouse lines have C57bl/6 background and were PCR-genotyped before use. Only male mice (2-3 months old) were used for *in vivo* behavioral assays.

In addition, adult male and female rhesus monkeys (*Macaca* *mulatta*, 11-18 years, 7.2-13.9 kg), were used for scratching behavior study. Monkeys were individually housed in species-specific and climate-controlled rooms on a 12/12-h light/dark cycle. Their daily diet consisted of approximately 22-30 biscuits (Purina Monkey Chow; Ralston Purina Co., St. Louis, MO), fresh fruit, and water *ad libitum*. Monkeys were kept at an indoor facility accredited by the Association for Assessment and Accreditation of Laboratory Animal Care International (Frederick, MD, USA). All animal care and experimental procedures were conducted in accordance with the Guide for the Care and Use of Laboratory Animals as adopted and promulgated by NIH and approved by the IACUC of Wake Forest University. This study is reported in accordance with the ARRIVE guidelines for reporting experiments involving animals.

**Human subjects**

No-liver-disease controls and primary biliary cholangitis (PBC) patients with unknown itch status were recruited via the Duke Gastroenterology-Hepatology outpatient program, non-alcoholic fatty liver disease (NAFLD) clinical research program. Controls had normal liver histology, and PBC patients were diagnosed according to the European Association for the Study of Liver Disease criteria.^9^ The study population consisted of 27 women and 8 men, aged 24-65 years (average 44) for controls and 25 women and 0 men, aged 39-83 years (average 58) for PBC.

Primary biliary cholangitis (PBC) patients with known itch-phenotypic information were recruited at two clinical sites: Liver and Internal Medicine Unit of the Warsaw Medical University, Poland, and Department of Medicine 1, Gastroenterology, Hepatology, Pneumology and Endocrinology of the University Hospital of Erlangen, Germany. The diagnosis of PBC was made according to the European Association for the Study of Liver Disease criteria.^9^ Itch intensity was quantified at the time-point of blood drawing using a visual analogue scale ranging from 0 to 10 (0-3: no/mild itch, 3-6: moderate itch, 6-10: severe/worst imaginable itch).^10, 11^ The study population in Poland consisted of 27 women and 2 men, aged 36-75 years (average 55), and 10 women without itch, 9 women and 1 man with moderate itch, and 8 women and 1 man with severe itch. For study population in Germany, all patients were women, aged 29-64 years (average 51), and 11 patients without itch, 2 with moderate itch, and 6 with severe itch.

Study protocols were approved by the local medical institutional review boards, and all subjects provided written consent for their samples to be used. The serum supernatant was aliquoted and frozen until measurements were performed. Patients’ samples were used in anonymized manner for our study without any recourse to proprietary health information.

**Chemicals and antibodies**

LPA 18:1, egg-LPC (LPC, mixture of LPC species 14:0, 16:0, 16:1, 18:0, 18:1, and 18:2), LPC 14:0, LPC 16:0, LPC 18:0, and LPC 18:1 were purchased from Avanti Polar Lipids (Alabaster, AL). GSK1016790A, SB366791, HC030031, capsaicin, histamine, HC067047, PF8380, U73122, resiniferatoxin, U0126, tamoxifen, 4-hydroxy tamoxifen, α-naphthyl isothiocyanate (ANIT), isopentenyl pyrophosphate (IPP), glycerophosphorylcholine phosphodiesterase, choline oxidase, peroxidase, 3-(N-Ethyl-3-methylanilino)-2-hydroxypropanesulfonic acid sodium salt (TOOS), and 4-aminoantipyrine were purchased from Sigma (St. Louis, MO). E6446 was purchased from Bocsci INC (Shirley, NY), BIM46187 was purchased from Aobious (Gloucester, MA), Gallein, GIT27, ICI 204,448 and Yoda 1 were purchased from Tocris Bioscience (Minneapolis, MN), lysophospholipase was from Sekisui Diagnostics (Burlington, MA), and GsMTx4 was from Adooq Bioscience LLC (Irvine, CA). GSK205 was synthesized by the Small Molecule Synthesis Facility at Duke University.^12^ miR-146a-5p mimic (miR-146a) and miR-146a-5p negative control (scramble) were obtained from Thermo Fisher (Waltham, MA). LPA, LPCs, histamine, and GsMTx4 were dissolved in sterile normal saline (NS) and freshly made for use. miR-146a mimic and scramble were dissolved in nuclease-free water and freshly prepared before use. All other chemicals were dissolved in DMSO and further diluted until use.

Rabbit polyclonal anti-phospho-ERK, monoclonal anti-ERK, and polyclonal anti-phospho-MEK were obtained from Cell Signaling Technology (Danvers, MA). Rabbit polyclonal anti-TRPV1 was obtained from Neuromics (Edina, MN), polyclonal anti-TRPA1 from Novus Biologicals (Centennial, CO), and polyclonal anti-TRPV4 from Abcam (Cambridge, MA) from Sigma. 4′,6-diamidino-2-phenylindole (DAPI) was obtained from Sigma.

##### Behavioral assessment

Mice were shaved at the dorsal neck 1 d before experiments. Mice were allowed to acclimate to a Plexiglas chamber for at least 30 min before testing and received intradermal (i.d.) injection of 50 μl of LPA 18:1, LPC, LPC 14:0, LPC 16:0, LPC 18:0, LPC 18:1, miR-146a mimic, miR-146a scramble, Yoda 1 or vehicles through a 30-gauge needle (Becton Dickinson, Franklin Lakes, NJ) into the neck skin. After injections, mice were immediately placed back to the chamber, and the scratching behavior was recorded by a Panasonic video camera for 30 min. Hind limb scratching behavior directed toward the injected area at the nape of neck was observed.  A scratch bout is defined as one or more rapid back-and-forth hind paw motions directed toward and contacting the treated area, ending with licking or biting of the toes or placement of the hind paw on the floor. Behavioral analysis was conducted by observers blinded to genotype.

To investigate the effects of the selective inhibitors GSK205 or HC067047 for TRPV4, SB366791 for TRPV1, HC030031 for TRPA1, PF8380 for autotaxin, U0126 for MEK, IPP for TRPV3, GIT for TLR2/6, E6446 for TLR7/9, non-selective inhibitor GsMTx4 for PIEZO 1, and selective agonist ICI 204,448 for kappa opioid receptor, on LPA-, LPC- or miR-146a-induced scratching behaviors, mice received an intraperitoneal (i.p.) injection of 0.25 ml or an intrathecal (i.t., see approach below) injection of 5 µl of chemical solutions 15 min before pruritogen injections. Control animals received the same volume of vehicles.

To test whether LPC induces scratching behavior at the spinal cord level directly, a 30-gauge needle attached to 10 µl micro-syringe (Hamilton Co., Reno, NV) was inserted between L4 and L5 segments and tail flick upon i.t injection was considered a control for targeted delivery. LPC was injected into the subarachnoid space with a total volume of 5 μl at a constant rate of 10 µl/min. Scratching behavior was recorded for 30 min and behavioral analysis was performed as described above.

To examine whether i.d. injection of LPC induces pain-like behavior in mice, a mouse cheek model was used to differentiate itch from pain.^13^ In brief, 10 µl of LPC or miR-146a was administrated into the mouse cheek. Wiping (pain) and scratching (itch) behaviors were videoed for 30 min using a Panasonic camera. A bout of wiping was defined as a continuous wiping movement with a forepaw directing at the area of the injection area and a bout of scratching was defined as above.

To examine whether TRPV1-expressing sensory neurons contribute to LPC- or miR-146a-induced itch, we ablated the central terminals of TRPV1-expressing neurons by i.t. injection of 200 ng resiniferatoxin (RTX) into the L4/L5 subarachnoid space.^14^ Lumbar puncture was made with a 30G-needle and drugs at 5 µl of volume were delivered. Scratching behavior assay was started approximately 2 weeks after resiniferatoxin treatment.

Scratching behaviors in monkeys were performed as previously described.^15^ In brief, monkeys were seated in primate chairs and both lateral sides of upper part (i.e., the skin area over the *vastus lateralis muscle*) of their hindlimbs were shaved 1 d before experiments. The monkey’s hindlimb was held by another experimenter during the injection. A 30G-needle connected with a 50 μl microsyringe (Hamilton Co., Reno, NV) was placed almost flat against skin, bevel up; and then was inserted 1/8 inch into skin. 20 μl of histamine, LPC or miR-146a solution was slowly injected and was watched for a wheal to appear. Once the injection was completed, monkeys immediately returned to his/her home cage and their potential site-specific scratching activity was recorded for 15 min after injection. A scratch was defined as one brief scraping contact of the forepaw or hind paw on the skin surface area. Scratching activities were scored by individuals who were blinded to dosing conditions. Before collecting data, monkeys had been habituated with the injection procedure and experimenter for several times. It is noted that the tested chemicals were i.d. administered to the same subjects with at least one-week interval, starting vehicle first and then chemicals in randomized doses. Based on our prior experience, we have not observed tolerance to elicited scratching with this schedule for a well-known pruritogen histamine. In addition, this repeated injection schedule with tested chemicals did not result in any skin lesion. There was no significant difference in scratching numbers between male (n=7) and female (n=2) monkeys for tested chemicals.

Mouse model of cholestasis was induced by α-naphthyl-isothiocyanate (ANIT, dissolved in corn oil) administration via oral gavage for 5 consecutive days at 25 mg/kg. Control mice received the same volume of corn oil. Animals were habituated to the testing environment for 2 d before baseline testing. The scratching behavior was recorded by a Panasonic video camera for 1 h every day before daily ANIT treatment.

To test whether LPC induces pain behavior at the spinal cord level directly, LPC was i.t. injected into the subarachnoid space with a total volume of 5 μl at a constant rate of 10 µl/min. Mechanical pain behavior was assessed with electronic von Frey filaments (Ugo Basile, Italy). Animals were habituated to the testing environment daily for at least 2 days before baseline testing. Mice were placed on a 5×5-mm wire-mesh grid floor in individual compartments and allowed to adapt for 0.5 h prior to the von Frey test. The von Frey filament was then applied to the middle of the plantar surface of the hind paw and the withdrawal responses following the stimulation were measured 3 times and averaged. Data on mechanical threshold was express as % of change.

**Cell culture and transfection**

HEK293 cells (ATCC^®^ CRL-1573) were cultured on poly-D-lysine coated coverslips in 24-well plate containing DMEM media with high-Glucose (Gibco, Gaithersburg, MD) supplemented with 10% fetal bovine serum (FBS, HyClone Laboratories, Logan, UT) and 100 U/ml of penicillin/streptomycin (Gibco, Gaithersburg, MD). Cell cultures were maintained with 5% of CO_2_ in a humidified incubator at 37 °C. HEK cells were transfected with rat or human wild type or mutant TRPV4 channels with EGFP or YFP coupled to their C-termini (1.5 µg of plasmid for excised patch clamp and Ca^2+^ imaging and 200 ng for single-channel experiments). The jetPEI™ Polyplus transfection reagent (Polyplus Transfection, New York, NY) for electrophysiology or Lipofectamine^TM^ 2000 Reagent (Invitrogen) for Ca^2+^ imaging were used for transfections per manufacturer’s instructions as previously described.^4, 16^ To investigate whether miR-146a can directly activate TRPV1 channels or indirectly via TLRs, HEK293 cells were transfected with rTRPV1 or co-transfected rTRPV1 with rTLR7, rTLR2, or rTLR6 (Addgene, [Watertown, MA](https://www.google.com/search?rlz=1C1CHZL_enUS703US703&q=Watertown,+Massachusetts&stick=H4sIAAAAAAAAAOPgE-LSz9U3yEsrNzQ0UOIAsUviLcq1jDLKrfST83NyUpNLMvPz9POL0hPzMqsSQZxiq4zUxJTC0sSiktSiYoWc_GSw8CJWifBEoEhJfnmejoJvYnFxYnJGaXFqSUnxDlZGAGsfOmNqAAAA&sa=X&ved=2ahUKEwius9WIh8jqAhVSPawKHa8nAhwQmxMoATAnegQICBAD)) for Ca^2+^ imaging assay. Control cells were transfected with GFP or YFP.

Primary mouse keratinocytes were cultured following previous protocol.^5^ The epidermis from the back skin of newborn WT or keratinocyte-*Trpv4* cKO mice was separated from the dermis by floating the skin on 0.25% trypsin (Gibco) for 14-18 h at 4 °C. Basal keratinocytes were separated from the cornified sheets by filtration through a 70 μM cell strainer. Keratinocytes were plated on collagen I-coated dishes or glass coverslips and grown in EME media (Gibco) containing 8% chelex (Bio-rad, Hercules, CA)-treated FBS with the final Ca^2+^ adjusted to 0.05 mM, bovine pituitary extract (50 µg/ml), epidermal growth factor (5 ng/ml), and 1x antibiotics/antimycotics (Gibco) in a humidified incubator with 5% CO_2_ at 37 °C for 5-7 days until use. To knockdown *Trpv4* in isolated keratinocytes from newborn keratinocyte-*Trpv4* cKO mice, cells were treated with 4-OH tamoxifen at 500nM for 72 h.

Primary human keratinocytes were cultured as previously described.^5^ In brief, surgically discarded foreskin samples, obtained from Duke Children's Hospital in accordance to institutionally approved IRB protocol, were incubated with Dispase (Gibco, 4 U/ml) for 12-16 h at 4°C followed by 0.05% trypsin (Gibco) for 10-20 min at 37°C. Cells were maintained in keratinocyte serum-free media (Invitrogen) with 5% CO_2_ at 37°C and used at passage 2-3.

Primary mouse sensory neurons were cultured following previous protocol.^4^ DRGs from 2-3 weeks old male WT, *Trpv1* KO, and *Trpa1* KO mice were dissected and digested with 1 mg/mL collagenase (Worthington, CSL1) and 5 mg/mL dispase (Invitrogen) for 1 h, then triturated. The resulting cell suspension was filtered through a 70 μm cell strainer (BD Biosciences, Bedford, MA) to remove debris. Neurons were cultured in DH10 medium (1:1 DMEM:Hams-F12, Invitrogen) with 10% FBS (Sigma), 100 U/mL penicillin and 100 μg/mL streptomycin (Gibco) and 50 ng/mL nerve growth factor (NGF; USBiological) on coverslips coated with poly-D-lysine and laminin (Invitrogen), and incubated with 5% CO_2_ at 37 °C. Ca^2+^ imaging was performed next day after culture.

**Modeling of LPC binding to TRPV4 channels**

The TRPV4 structure from *Xenopus tropicalis* (PDB file 6BBj, ^17^) was used within the Schrodinger software package. Within Schrodinger, the protein preparation wizard within the Maestro Molecular Modeling Interface was run, and a conformer library of LPC 18:1, was generated using the ligand preparation tool. Within the Schrodinger software tool, the TRPV4 protein structure was minimized using the optimized potentials for liquid simulations model force field with standard parameters (OPLS, version 3e).^18^ The receptor grid generation was centered on residue K750 using the maximum 36 Angstrom distance. Ligand-receptor docking was performed using the Glide software platform. ^19^

**Site-directed mutagenesis of TRPV4 channels**

TRPV4 sequences of multiple species were aligned using Clustal Omega program. Site-directed mutagenesis to generate point mutations in rat and human TRPV4 channels were carried out using Phusion DNA polymerase enzyme ^20-22^, with final sequencing to check for presence of the induced mutation. We introduced the following mutations into the rTRPV4 or hTRPV4 channel: R746C, R746G, R746D, K754G, R757G, R774G, and W776G.

**In vitro and ex vivo Ca^2+^ imaging**

Routine procedures were followed for Ca^2+^ imaging in cultured DRG sensory neurons, epidermal keratinocytes, and HEK cells.^4, 5^ Ca^2+^ imaging of cultured cells in response to chemical solutions was conducted after loading with 5 μM Fura2-AM (Invitrogen) for 45 min following a ratiometric Ca^2+^-imaging protocol with 340/380-nm blue light for dual excitation. Ratios of emissions were acquired at 0.5 Hz. ΔR/R_0_ was determined as the fraction of the increase of a given ratio over baseline ratio divided by baseline ratio. To investigate the effects of the TRPV4 inhibitors GSK205 or HC067047, the PLC inhibitor U73122, the Gα_q_ G Protein inhibitor BIM46187, the Gβγ G protein inhibitor Gallein, the TRPV1 inhibitor SB366791, the TRPA1 inhibitor HC030031, the TRPV3 inhibitor IPP, the PIEZO 1 inhibitor GsMTx4, on LPA-, LPC-, miR-146a, or Yoda 1-induced Ca^2+^ influx, cells were incubated with the inhibitors for 15 min before stimulation. Control cells received vehicles.

A previously established method was followed for *ex vivo* Ca^2+^ imaging of DRG explants.^2^ Intact DRGs (L4 or L5) were isolated from naïve male or female Pirt-GCaMP3 mice (2-3 months old) and equilibrated in artificial cerebrospinal fluid (ACSF) bubbled with 95% O_2_/5% CO_2_ at room temperature. After 15 min, explants were placed in a dish with 2 ml of pre-oxygenated ACSF and imaged using a Zeiss 780 upright confocal microscope (Carl Zeiss AG, Oberkochen, Germany) with 20x water immersion objective and Z-stack approach at the 488 nm wavelength. Explants were stimulated by miR-146a or miR-146a scramble. After 15 min recording, capsaicin was then applied to identify whether the miR-146a responding neurons were TRPV1 positive. In addition, to examine whether inhibition of TRPV1 or TRPA1 ion channels attenuates miR-146a-induced Ca^2+^ signal, explants were pretreated with the TRPV1 inhibitor SB366791 or the TRPA1 inhibitor HC030031 during the 15 min sample equilibration. Ca^2+^ fluorescence intensity was determined using the ImageJ software (NIH, Bethesda, MD, USA). For each neuron, the pixel intensity (F_t_) was assessed for each frame and the pixel intensity recorded from the first 20 frames was taken to determine the average baseline value (F_0_). Ca2+ signal amplitudes are presented as ΔF/F_0_, which is the ratio of fluorescence difference (F_t_-F_0_) to baseline (F_0_).

**Electrophysiology**

Currents were recorded using the inside-out configuration of the patch-clamp technique.^23^ Solutions were changed with a RSC-200 rapid solution changer (Molecular Kinetics). GSK1016790A was prepared in DMSO at 15.25 mM for the stock, which was kept at -20 ºC and diluted to 1 µM in recording solution for application to membrane patches. LPC 18:1 was prepared in DMEM-BSA 0.1% at 10 mM for stock solutions, kept at -70 ºC and diluted in recording solution. The recording solutions contained (in mM): 130 NaCl, 3 HEPES (pH 7.2) and 1 EDTA for the bath and 130 NaCl, 3 HEPES (pH 7.2) and 5 CaCl_2_ in the pipette. Experiments were performed at room temperature. Mean current values in response to GSK1016790A or LPC 18:1 were measured after channel activation had reached the steady-state (~3 min). Currents were obtained using voltage protocols where the holding potential was 0 mV and 10 mV steps from -120 to 120 mV or from -60 to +60 mV for 100 ms, to 0 mV. Borosilicate glass was used for pipette fabrication (5 MΩ). Currents were low-pass filtered at 2 kHz and sampled at 10 kHz with an EPC 10 amplifier (HEKA Elektronik) and were plotted and analyzed with Igor Pro (Wavemetrics Inc.).

For single-channel recordings, Borosilicate glass 30 MΩ pipettes were used. Recordings were obtained at +60mV by acquiring several traces of 1-3s duration. The effect of GSK1016790A or LPC 18:1 on single TRPV4 channels was studied in inside-out. Currents were filtered at 2 kHz and sampled at 5 kHz. Patches containing only one channel activated by different compounds were identified as those that did not contain overlapping opening events. Single-channel openings and closures were identified with the half-threshold crossing technique.^16^ The channel open probability was calculated as the sum of the total open time divided by the sweep duration. Dwell times and amplitude histograms in the closed or open states were collected in logarithmic time histograms according to the Sine-Sigworth transformation.^24^ Sums of three or two exponential components were fitted to histograms using a least-squares algorithm.

**In vitro interaction assays of LPC-TRPV4**

Surface proteins were obtained from HEK293 cells transiently expressing rTRPV4-EGFP channels using the Pierce Cell Surface Isolation kit (Pierce Biotechnology, Rockford, IL) following the manufacturer’s instructions. Overlay assays were performed as previously described.^16^ In brief, LPC 18:1 was spotted (200 pmol per spot) onto a nitrocellulose membrane (GE Healthcare, Pittsburgh, PA) and then blocked with 1% fatty acid-free BSA (Calbiochem) and 6% fat-free dried milk in PBS. Membranes were then incubated with the surface protein solutions and exposed to anti-GFP antibody (Sigma) diluted 1:1000 in 3% fat-free dried milk in PBST (with 0.1% of Tween). Membranes were incubated with horseradish peroxidase-conjugated secondary anti-rabbit antibody (Cell Signaling Technology) diluted 1:7500 in 6% fat-free dried milk in PBST. The binding of rTRPV4-EGFP and TRPV4-R746D-EGFP to the lipid-containing spots was visualized by chemiluminescence by exposing the blot for 15 min (Amersham Bioscience, Piscataway, NJ). Semi-quantitative densitometric analysis was done using ImageJ (NIH) and expressed as relative protein levels of TRPV4 bound to each spot.

##### Western Blot

Routine procedures were followed.^4, 5^ Briefly, cultured keratinocytes and dissected dorsal neck skin (0.5×0.5 cm, the area that received the treatment) were protein-extracted in radioimmunoprecipitation assay (RIPA, Sigma) buffer and electroblotted to polyvinylidene fluoride (PVDF)  membranes after gel separation of proteins in a 4-15% polyacrylamide gel (Bio-Rad). Membranes were blocked with 5% BSA in TBST, and incubated with primary antibodies rabbit anti-pERK or anti-ERK (both at 1:2000) followed by secondary antibody (anti-rabbit peroxidase-conjugated, 1:5000; Jackson ImmunoResearch), and chemiluminescence substrate (ECL-Advance, GE Healthcare). Immunoblot band intensity was quantitated using the software Image J and ERK served as a control for pERK expression.

**Immunohistochemistry and morphometry analysis**

Routine procedures were followed.^4^ Briefly, mice were perfused transcardially with 0.01 M PBS followed by ice-cold 4% paraformaldehyde (PFA, Sigma). Cervical spinal cord, TGs and cervical DRGs, and dorsal neck skin were dissected and post-fixed in 4% PFA overnight, cryoprotected in 20% sucrose (48 h) and sectioned on a cryostat (30 μm for spinal cord, 12 μm for TG and DRG and neck skin). Sections were blocked with 5% normal goat serum (Jackson), and incubated overnight with primary antibodies: rabbit anti-TRPV1 (1:5000), TRPA1 (1:200), TRPV4 (1:300) or p-MEK (1:200). Immunodetection was accomplished with secondary antibodies (AlexaFluor 594-conjugated goat anti-rabbit) for 2h, and cover-slipped with Vectashield (Vector). DAPI (1 µg/ml) was used for counterstaining with p-MEK in skin sections. Digital micrographs were acquired using a BX61 Olympus upright microscope with a high-resolution ORCA-Spark camera (Hamamatsu) and with constant acquisition/ exposure settings, using CellSens Dimension software (Olympus). 4-6 sections were analyzed per mouse. TG and DRG neurons were identified by morphology. The cutoff density threshold was determined by averaging the density of three neurons per section that were judged to be minimally positive, using ImageJ software. All neurons for which the mean density exceeded the threshold >25% were judged as positive. Positive cells were expressed as % of total counted TG neurons. The labeling density of TRPV1 in spinal cord was measured using the integrated density algorithm of Image J.

**Measurement of released vesicles and extracellular miR-146a from cultured keratinocytes or sera**

Medium for the cultured keratinocytes was replaced with serum-free medium 2 h before LPC stimulation. Fifteen minutes after LPC, the supernatant of the cells was harvested and subjected to two steps of centrifugation (i) 300 g for 5 min to eliminate remaining cells, (ii) 16,000 g for 30 min to eliminate cell debris and apoptotic bodies. Finally, cell-free supernatants were further purified using a Vesicular Isolation kit (Invitrogen) according to the manufacturer's instructions, with final product resuspended in ice-cold PBS. Total RNA extraction was then carried out using a Total RNA Isolation kit (Invitrogen). Enrichment for small RNAs was carried out by sequential increase in ethanol concentration and passing through glass-fiber filters. RNase free water was used to elute small RNAs in the final elution step. For human PBC sera or sera from ANIT-treated mice, RNA was isolated using Qiagen miRNeasy Plasma/Serum kit. cDNA synthesis from extracted RNAs was performed according to manufacturer’s instructions (qSTAR miRNA kit, Origene). For qPCR, the stem-loop oligonucleotides specific for the following miRs are as follows: miR-146a-5p (GAGAACTGAATTCCATGGG), miR-let-7b (GAGGTAGTAGGTTGTGTGG), miR-125b-1 (CCCT GAGACCCTAACTTG), miR-203 (GTGGTTC TTGACAGTTCAAC), and miR-16-5p (AGCAGCAC GTAAATATTGGC). Primers were purchased from Integrated DNA Technologies company. qPCR reactions for each sample were run in triplicates, including no-template controls. MiR-16-5p was selected as a control due to the relative constancy of its expression in various cultured cell lines.^25^ qPCR for this miR was performed in tandem with target miRs to determine the optimal normalization procedure. To investigate the effects of the specific TRPV4 inhibitors GSK205 or HC067047 and the specific MEK inhibitor U0126 on LPC-induced extracellular release of miR-146a, cells were incubated with the inhibitors for 15 min before stimulation. Control cells received the same volume of vehicle.

Vesicular release from cultured keratinocytes was quantified by detecting acetylcholinesterase (AChE) activity in the extracellular release fluid. ^26, 27^ CBQCA Protein Quantitation Kit (Molecular Probes, [Eugene, OR](https://www.google.com/search?rlz=1C1CHZL_enUS703US703&q=Eugene,+Oregon&stick=H4sIAAAAAAAAAOPgE-LSz9U3MKowLDAtUuIAs3PTzLW0spOt9POL0hPzMqsSSzLz81A4VhmpiSmFpYlFJalFxYtY-VxL01PzUnUU_ItS0_PzdrAyAgCe_AtJWAAAAA&sa=X&ved=2ahUKEwjjg7742szqAhVPR60KHfA-Aw8QmxMoATAXegQIDBAD)) was used to assess the total protein amounts of each sample. Quantitation was carried out according to kit instructions (Fluorocet, Systems Biosciences, Palo Alto, CA). Briefly, vesicles were lysed to release esterase enzyme whose activity is measured using a florescence dye, excitation at 544 nm and emission at 590 nm. Fluostar Optima (BMG Labtech, Cary, NC) microplate reader was used to measure esterase activity. 500ng protein equivalent of input was used per well. To determine the contribution of Rab5a and Rab27a to LPC-induced miR-146a release, cells were pre-treated with the Rab5a (5’-GUAGAAUCAA GUUUCUAAUUCUGAA-3’, 5’-UUCAGAAUUAGAAACUUGAUUCUACCA-3’) or Rab27a (5’-AGCUAAAA CUGAGAGCUUCAAACAG-3’, 5’-CUGUUUGAAGCUCUCAGUUUUAGCUUA-3’) siRNA (IDT, [Coralville, IA](https://www.google.com/search?rlz=1C1CHZL_enUS703US703&q=Coralville,+Iowa&stick=H4sIAAAAAAAAAOPgE-LSz9U3MMnKyLVIUuIAsUsMDDK0tLKTrfTzi9IT8zKrEksy8_NQOFYZqYkphaWJRSWpRcWLWAWc84sSc8oyc3JSdRQ888sTd7AyAgBIlqg5WgAAAA&sa=X&ved=2ahUKEwi2v4Sbn9PqAhWRZd8KHdkHCjYQmxMoATAXegQIBxAD)) for 72h before stimulation. Control cells were treated with scramble siRNA.

**LPC measurement in sera and skin**

Blood and dorsal neck skin (~ 0.5 x0.5 cm) were harvested from ANIT- or control-treated mice at day 5. Blood was drawn via cardiac puncture and allowed to clot at room temperature for 20 min. After centrifugation at 2000xg for 10 min at 4°C, serum was collected and stored at -80 °C until use. After weighing, skin was cut into pieces and sonicated in methanol for 1 min at 4°C. After sonication, samples were centrifuged at 12000xg for 15min at 4°C and supernatant was collected and stored at -80 °C until analysis. Total levels of LPC in mouse serum and skin were determined by an enzymatic colorimetric method.^28^ In brief, 8 µl of samples were treated with lysophospholipase, glycerophosphorylcholine, phosphodiesterase, and choline oxidase. The resulting hydrogen peroxide generated was quantified using horseradish peroxidase and TOOS reagent. The absorbance was measured by microplate reader (Molecular Devices, San Jose, CA). Total level of LPC was detected at around 1 mM in sera of control mice. Total levels of LPC in serum of no-liver-disease controls (~218 µM) and PBC patients from Duke Gastroenterology-Hepatology outpatient program, non-alcoholic fatty liver disease (NAFLD) clinical research program were also determined by this enzymatic colorimetric method as described above.

Serum levels of LPC in PBC patients from two clinical sites, Liver and Internal Medicine Unit of the Warsaw Medical University, Poland, and Department of Medicine 1, Gastroenterology, Hepatology, Pneumology and Endocrinology of the University Hospital of Erlangen, Germany, were determined by the AbsoluteIDQ™ p180 kit (Biocrates, Life Sciences AG, Innsbruck, Austria) following the manufacturer’s instructions. The assay allows simultaneous quantification of 188 metabolites out of 10 μl serum, including 14 species of LPC: 14:0, 16:0, 16:1, 17:0, 18:0, 18:1, 18:2, 20:3, 20:4, 24:0, 26:0, 26:1, 28:0, and 28:1. In brief, after 10 μl internal standards or 10 μl serum were added into the filter wells of a 96-well plate, the wells were dried by nitrogen, and 50 μl of 5% phenyl-isothiocyanate solution was added into each well for derivatization. After incubation, the wells were dried again, and 300 μl of methanol containing 5 mM ammonium acetate were added into each well to extract the metabolites. The extract was then centrifuged into the collection wells, and each well was diluted with 300 μl of the running solvent (a proprietary mixture provided by Biocrates). The flow injection analysis-tandem mass spectrometry (FIA-MS/MS) technique was used to detect 14 species of LPC. Using electrospray ionization in positive mode, samples were introduced directly into a Xevo TQ-S triple quadrupole mass spectrometer (Waters) operating in the Multiple Reaction Monitoring (MRM) mode. MRM transitions (compound-specific precursor to product ion transitions) for each analyte and internal standard were collected over the appropriate retention time. The FIA-MS/MS data were analyzed using Biocrates MetIDQ™ software. Internal standards and quality control samples of the p180 Kit were utilized to benchmark the quality of the assay and the robustness of the data. For all analyzed LPC species, data were expressed in relative concentrations. Of note, LPC 14:0 data was not shown since it was below the lower limit of detection. Total level of 14 LPC species as above was detected at around 155 µM in sera of PBC patients without itch.

**Quantitative real-time PCR**

Total RNA from TGs or cervical DRGs was prepared using Directzol RNA kit (Zymo Research) following the manufacturer’s instructions. RNA was aliquoted and stored at -80 °C until use. 1 µg of total RNA was reverse transcribed using SuperScript III Reverse Transcriptase (Invitrogen). Real-time PCR was performed with equal amounts of cDNA in the GeneAmp 7700 sequence detection system (Applied Biosystems) using QuantiTect SYBR Green PCR Kit (Qiagen). The ∆∆Ct method was used for relative quantification of gene expression. Primers were synthesized by Integrated DNA Technologies and their sequences are: internal control ß-tubulin (forward: 5’-CCTG CCTTTTCGTCTCTAGC CGC-3’, reverse: 5’-GCTGATGACCTCCCA GAACTTGGC-3’) and TRPV4 (forward: 5’-GTGGGCAAGAGCTCAGATGGCACTC-3’, reverse: 5’-CCACCGAGG ACCAACGATCCCTAC G-3’).

**Supplementary References**

1. Liedtke W, Tobin DM, Bargmann CI, et al. Mammalian TRPV4 (VR-OAC) directs behavioral responses to osmotic and mechanical stimuli in Caenorhabditis elegans. Proc Natl Acad Sci U S A 2003;100 Suppl 2:14531-6.

2. **Kim YS**, **Chu Y**, Han L, et al. Central terminal sensitization of TRPV1 by descending serotonergic facilitation modulates chronic pain. Neuron 2014;81:873-887.

3. Moore C, Cevikbas F, Pasolli HA, et al. UVB radiation generates sunburn pain and affects skin by activating epidermal TRPV4 ion channels and triggering endothelin-1 signaling. Proc Natl Acad Sci U S A 2013;110:E3225-34.

4. Chen Y, Kanju P, Fang Q, et al. TRPV4 is necessary for trigeminal irritant pain and functions as a cellular formalin receptor. Pain 2014;155:2662-72.

5. Chen Y, Fang Q, Wang Z, et al. Transient Receptor Potential Vanilloid 4 Ion Channel Functions as a Pruriceptor in Epidermal Keratinocytes to Evoke Histaminergic Itch. J Biol Chem 2016;291:10252-62.

6. Agarwal N, Offermanns S, Kuner R. Conditional gene deletion in primary nociceptive neurons of trigeminal ganglia and dorsal root ganglia. Genesis 2004;38:122-9.

7. **Dankort D**, **Curley DP**, Cartlidge RA, et al. Braf(V600E) cooperates with Pten loss to induce metastatic melanoma. Nat Genet 2009;41:544-52.

8. **Van Keymeulen A**, **Rocha AS**, Ousset M, et al. Distinct stem cells contribute to mammary gland development and maintenance. Nature 2011;479:189-93.

9. EASL Clinical Practice Guidelines: The diagnosis and management of patients with primary biliary cholangitis. J Hepatol 2017;67:145-172.

10. Jacoby A, Rannard A, Buck D, et al. Development, validation, and evaluation of the PBC-40, a disease specific health related quality of life measure for primary biliary cirrhosis. Gut 2005;54:1622-9.

11. Raszeja-Wyszomirska J, Wunsch E, Krawczyk M, et al. Assessment of health related quality of life in polish patients with primary biliary cirrhosis. Clin Res Hepatol Gastroenterol 2016;40:471-9.

12. Kanju P, Chen Y, Lee W, et al. Small molecule dual-inhibitors of TRPV4 and TRPA1 for attenuation of inflammation and pain. Sci Rep 2016;6:26894.

13. Shimada SG, LaMotte RH. Behavioral differentiation between itch and pain in mouse. Pain 2008;139:681-7.

14. Mishra SK, Hoon MA. Ablation of TrpV1 neurons reveals their selective role in thermal pain sensation. Mol Cell Neurosci 2010;43:157-63.

15. Lee H, Ko MC. Distinct functions of opioid-related peptides and gastrin-releasing peptide in regulating itch and pain in the spinal cord of primates. Sci Rep 2015;5:11676.

16. Morales-Lázaro SL, Llorente I, Sierra-Ramírez F, et al. Inhibition of TRPV1 channels by a naturally occurring omega-9 fatty acid reduces pain and itch. Nat Commun 2016;7:13092.

17. Deng Z, Paknejad N, Maksaev G, et al. Cryo-EM and X-ray structures of TRPV4 reveal insight into ion permeation and gating mechanisms. Nat Struct Mol Biol 2018;25:252-260.

18. Harder E, Damm W, Maple J, et al. OPLS3: A Force Field Providing Broad Coverage of Drug-like Small Molecules and Proteins. J Chem Theory Comput 2016;12:281-96.

19. Friesner RA, Banks JL, Murphy RB, et al. Glide: a new approach for rapid, accurate docking and scoring. 1. Method and assessment of docking accuracy. J Med Chem 2004;47:1739-49.

20. **Salazar H**, **Jara-Oseguera A**, **Hernandez-Garcia E**, et al. Structural determinants of gating in the TRPV1 channel. Nat Struct Mol Biol 2009;16:704-10.

21. Rosenbaum T, Gordon SE. Dissecting intersubunit contacts in cyclic nucleotide-gated ion channels. Neuron 2002;33:703-13.

22. Hsieh PC, Vaisvila R. Protein engineering: single or multiple site-directed mutagenesis. Methods Mol Biol 2013;978:173-86.

23. Hamill OP, Marty A, Neher E, et al. Improved patch-clamp techniques for high-resolution current recording from cells and cell-free membrane patches. Pflugers Arch 1981;391:85-100.

24. Sigworth FJ, Sine SM. Data transformations for improved display and fitting of single-channel dwell time histograms. Biophys J 1987;52:1047-54.

25. **Schwarzenbach H**, **da Silva AM**, Calin G, et al. Data Normalization Strategies for MicroRNA Quantification. Clin Chem 2015;61:1333-42.

26. Gupta S, Knowlton AA. HSP60 trafficking in adult cardiac myocytes: role of the exosomal pathway. Am J Physiol Heart Circ Physiol 2007;292:H3052-6.

27. Malik ZA, Liu TT, Knowlton AA. Cardiac Myocyte Exosome Isolation. Methods Mol Biol 2016;1448:237-48.

28. Kishimoto T, Soda Y, Matsuyama Y, et al. An enzymatic assay for lysophosphatidylcholine concentration in human serum and plasma. Clin Biochem 2002;35:411-6.

**(Author names in bold designate shared co-first authorship)**


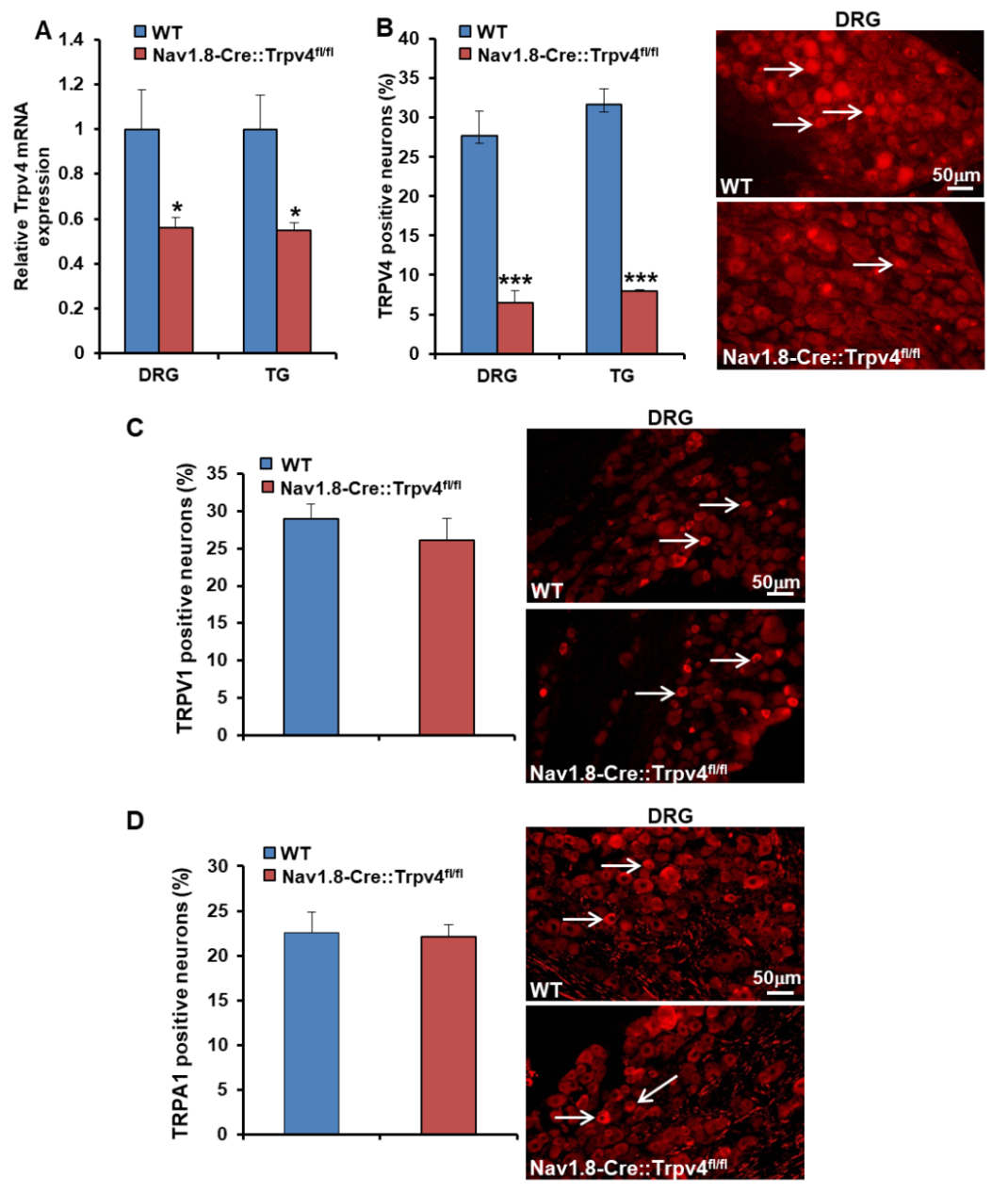


**Supplementary Figure 1. Depletion of *Trpv4* mRNA and protein in dorsal root ganglion (DRG) and trigeminal ganglion (TG) of Nav1.8-Cre::Trpv4^fl/fl^ mice.**

(*A*) qRT-PCR shows that *Trpv4* mRNA was significantly reduced in Nav1.8-Cre::Trpv4^fl/fl^ mice. ^*^p<0.05 vs WT, two-tailed t-test, n=4-5 mice/group.

(*B-D*) Immunostaining with their respective, specific antibodies shows reduced TRPV4-(B), but unchanged TRPV1(C)- or TRPA1(D)-expressing neurons, in Nav1.8-Cre::Trpv4^fl/fl^ mice. Arrows represent TRP-positive neurons.  ^***^p<0.001 vs WT, two-tailed t-test, n=4 mice/group (>1200 total neurons counted/group).


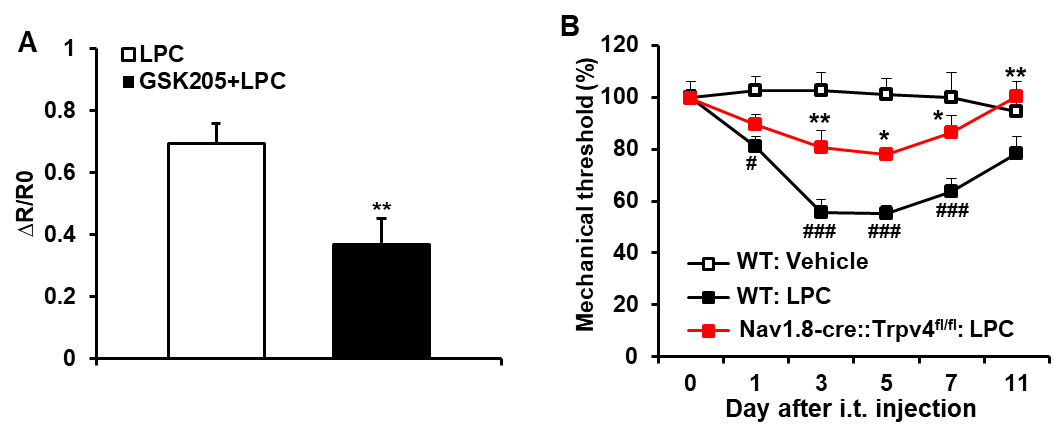


**Supplementary Figure 2. Intrathecal (i.t.) injection of LPC induces mechanical pain via activation of TRPV4-expressing dorsal root ganglion (DRG) sensory neurons.**

(*A*). Inhibition of TRPV4 with its selective inhibitor GSK205 (10 µM) reduced LPC (10 µM)-induced Ca^2+^ signal in cultured DRG neurons. **p<0.01 vs. LPC, two-tailed t test. N≥250 cells recorded/treatment.

(*B*). Single i.t. injection of LPC (15 µg/5 µl) induced long-lasting pain, as evidenced by reduced mechanical withdrawal thresholds. Pain behavior was attenuated in sensory neuron-*Trpv4* cKO (Nav1.8-Cre::Trpv4^fl/fl^) . ^#^p<0.05 and ^###^p<0.001 vs. WT: Vehicle (normal saline), and *p<0.05 and **p<0.01 vs. WT: LPC, two-way ANOVA with Tukey’s post-hoc test. N=7-8 mice/group.


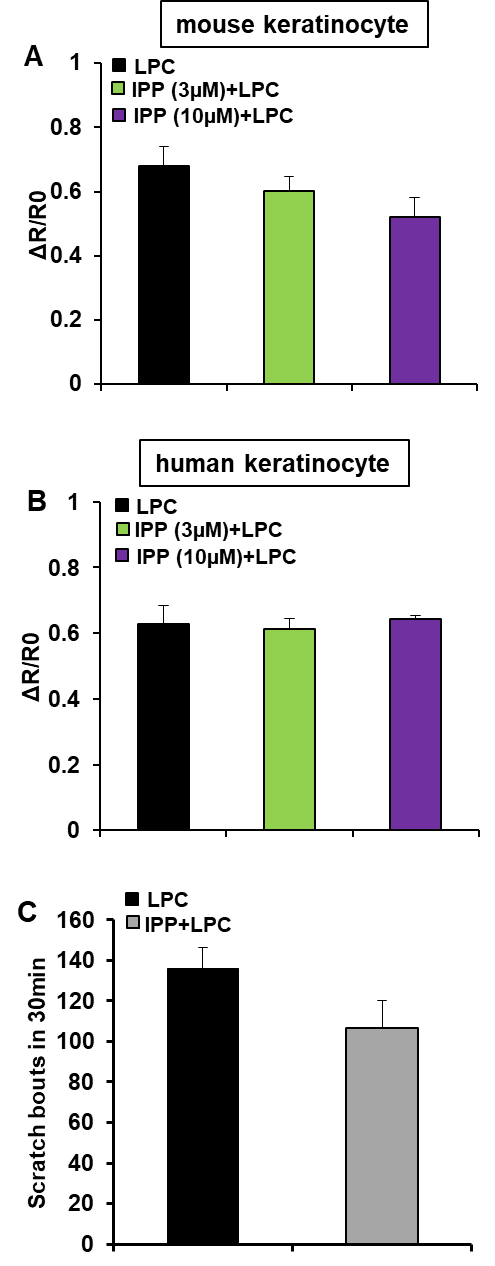


**Supplementary Figure 3. Keratinocyte-TRPV3 is not involved in LPC-induced itch.**

(*A-B*): LPC (10 μM)-induced Ca^2+^ signal in cultured mouse (A) or human (B) keratinocytes was not significantly influenced by inhibition of TRPV3 with selective inhibitor IPP. One-way ANOVA with Tukey’s post-hoc test, n≥180 cells recorded/treatment.

(*C*): i.d. injection of LPC (500µg)-induced itch was not significantly attenuated in mice pre-treated with TRPV3 inhibitor IPP (10 mg/kg, i.p.). Two-tailed t test. N=13 mice for LPC and 5 for IPP+LPC.


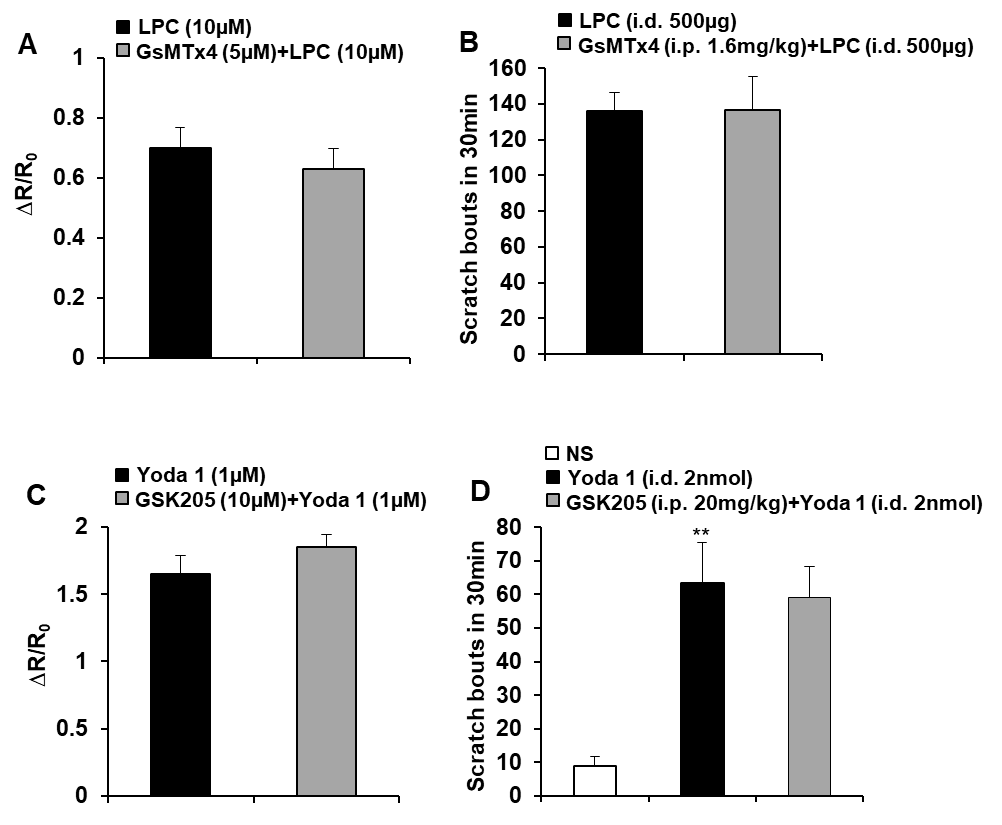


**Supplementary Figure 4. Piezo-1 is not involved in LPC-TRPV4-induced itch.**

(*A-B*): LPC-induced Ca^2+^ signal in cultured mouse keratinocytes (A) and scratching behavior (B) were not significantly influenced by PIEZO-1 inhibitor GsMTx4. Two-tailed t test, n≥180 cells recorded/treatment (A) and n=13 mice for LPC and 5 for GsMTx4+LPC (B).

(*C-D*): Activation of Piezo-1 with the selective agonist Yoda 1 caused Ca^2+^ signal in cultured mouse keratinocytes (C) and scratching behavior (D), which were not significantly affected by pretreatment with TRPV4 selective inhibitor GSK205. Two-tailed t test, n≥188 cells recorded/treatment (C) and One-way ANOVA with Tukey’s post-hoc test, n=4-5 mice/group, **p<0.01 vs NS (normal saline) (D).


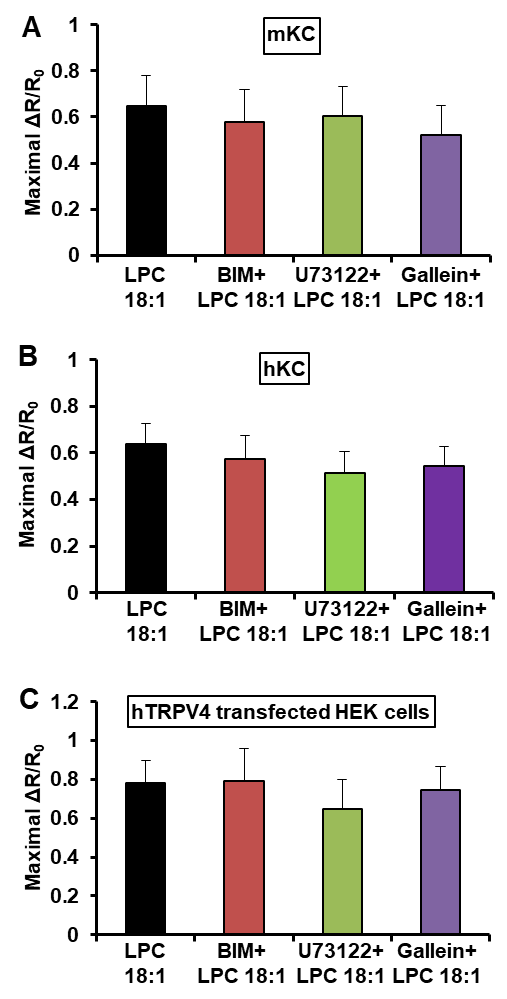


**Supplementary Figure 5. Lack of evidence for GPCR-signaling upstream of TRPV4 in Ca^2+^ influx.**

Ca^2+^ influx in cultured mouse (*A*) and human (*B*) keratinocytes (KC) and in HEK cells transfected with hTRPV4 (*C*), induced by LPC 18:1 (10 µM), was not significantly altered by Gα_q_ inhibitor BIM46187 (BIM), phospholipase C inhibitor U73122, or Gβγ inhibitor Gallein (all at 10 µM). One-way ANOVA with Tukey’s post-hoc test. n≥140 cells recorded/treatment.


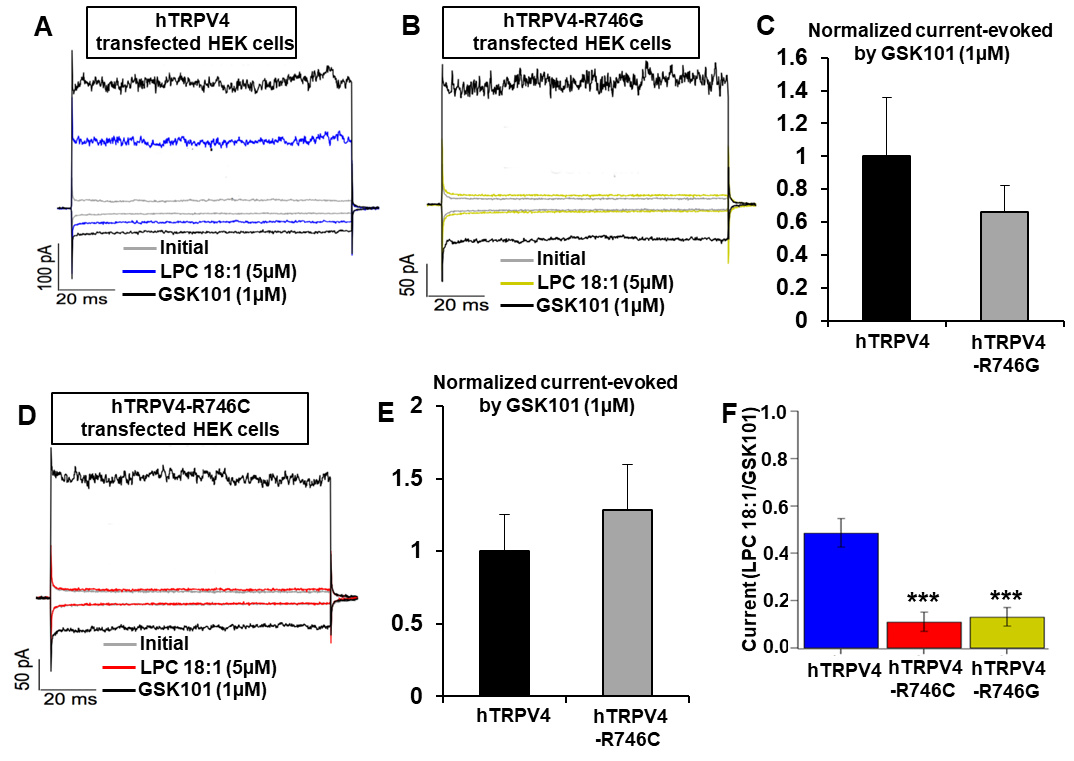


**Supplementary Figure 6. Electrophysiology findings of hTRPV4 with mutations at R746 reiterate its critical relevance for channel activation by LPC 18:1**

(*A-E*) Representative currents for hTRPV4 (A), hTRPV4-R746G (B), and hTRPV4-R746C (D) channels: Currents were recorded in the absence of agonist (grey), in the presence of 5 µM LPC 18:1 (blue, yellow and red, respectively) or in the presence of 1 µM GSK101 (black) at -60 and 60 mV. (C) Inert glycine mutation R746G of hTRPV4 responds to chemical activation with GSK101. Note lack of significant differences between groups, two-tailed t test, n=4 cells recorded for TRPV4(WT), left-hand black bars, average current pegged to "1", n=5 cells for TRPV4(R746G), right-hand grey bars. (E) Human genomic polymorphism mutation R746C of hTRPV4 responds to chemical activation with GSK101. Note lack of significant differences between groups, twp-tailed t-test, n=9 cells recorded for both channel isoforms; TRPV4(WT), left-hand black bars, average current pegged to "1", TRPV4(R746C), right-hand grey bars.

(*F*) There was a significant reduction of currents in hTRPV4-R746C or hTRPV4-R746G transfected HEK cells when activating with 5 µM LPC 18:1 (average activation was 48 ± 6% for WT-hTRPV4, 11 ± 4% for hTRPV4-R746C, and 13 ± 4% for hTRPV4-R746G). Data were normalized to activation obtained with 1 µM GSK101. ***p<0.001 vs. hTRPV4, One-way ANOVA with Tukey’s post-hoc test. N=5-10 cells/condition.


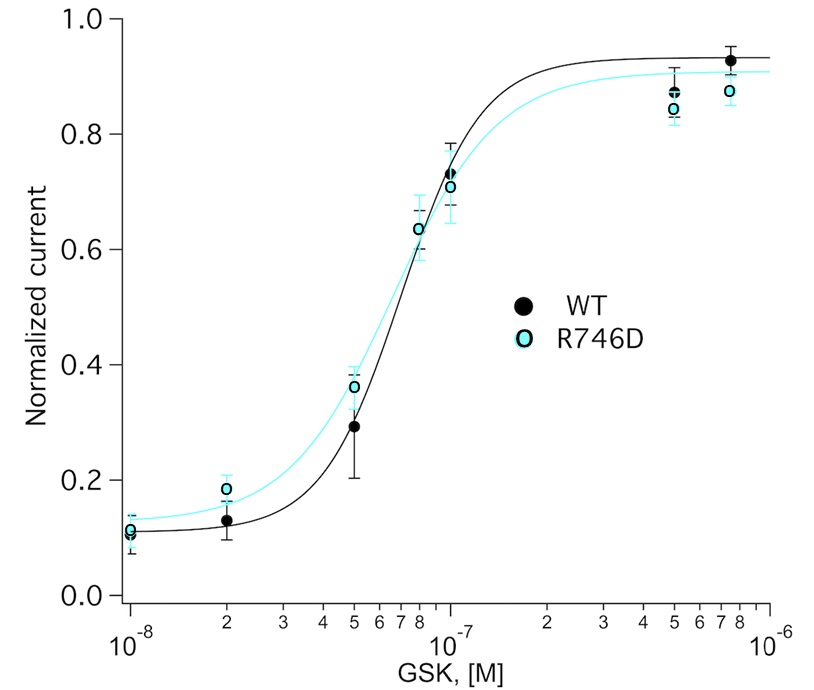


**Supplementary Figure 7. Dose-response curve for activation with GSK101 at +100 mV in the inside-out configuration for rTRPV4 (WT, filled symbols, black) vs rTRPV4 (R746D, empty symbols, turquoise) transfected HEK cells.** Note that there was no significant difference between WT and the R746D mutant. Smooth curve is a fit with the Hill equation (rTRPV4(WT) EC_50_=70nM and Hill coefficient=3.2; rTRPV4(R746D) EC_50_=66nM and Hill coefficient=2.6). Due to seal instability a single GSK101 concentration was tested per membrane-patch and the current was normalized to the current activated with 1μM GSK101 in the same patch. Group data are represented as mean±SEM of 4-9 patches per concentration.


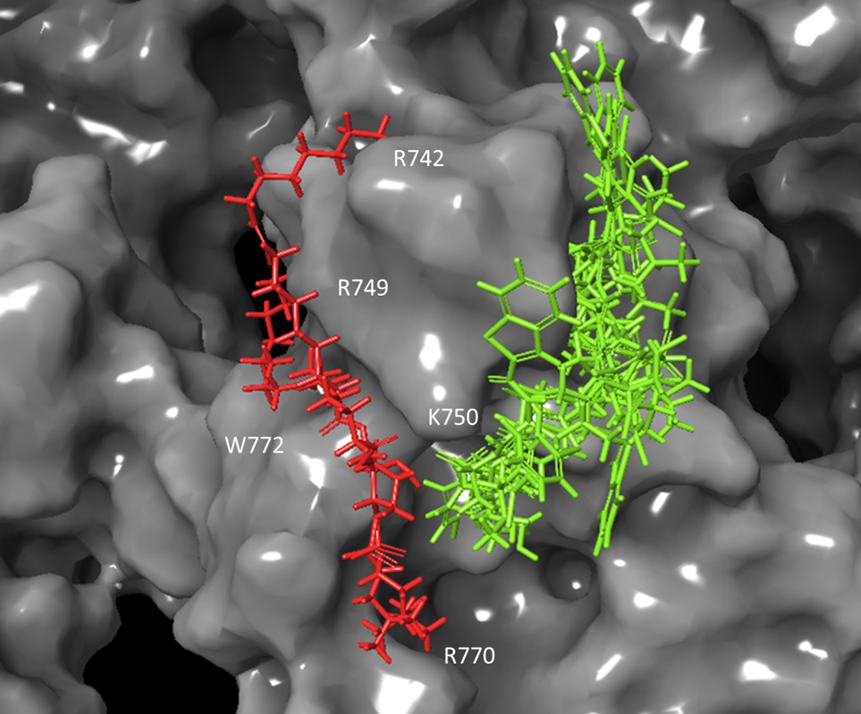


**Supplementary Figure 8. Overlay of all the predicted conformer binding poses of LPC 18:1 (left, red) and GSK 101 (right, green) relative to highlighted residues of xenopus TRPV4.**


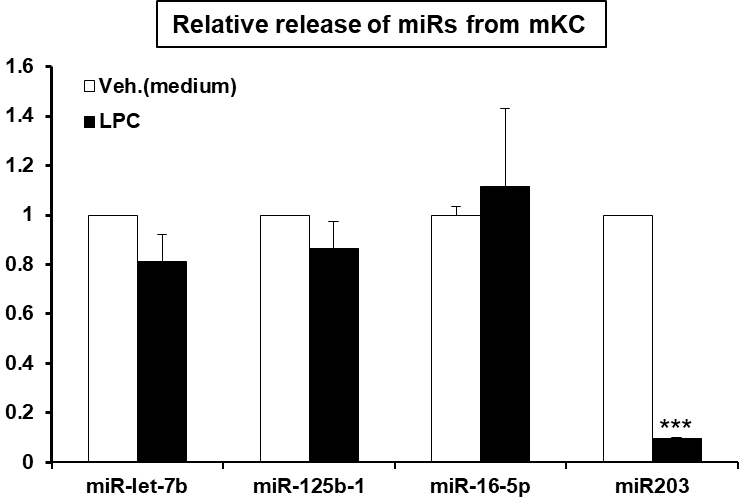


**Supplementary Figure 9. LPC stimulation (10 µm, 15min) did not increase the extracellular release of miR-let-7b, miR-125b-1, miR-16-5p, or miR-203 from cultured mouse keratinocytes (KC).** **p<0.001 vs. Veh. (medium), two tailed t -test was used. N=3 cultures/group (5-7 pups/culture).


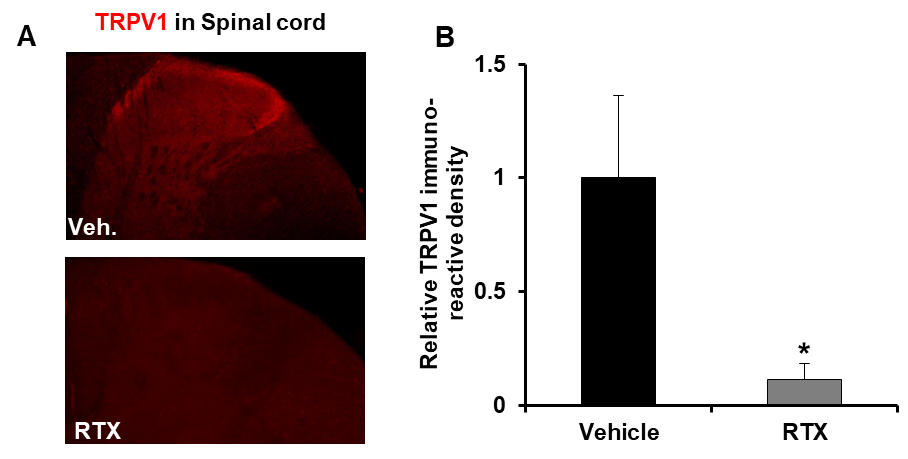


**Supplementary Figure 10. Elimination of TRPV1^+^ central nerve terminals in superficial layers of the spinal cord by i.t. injection of resiniferatoxin (RTX).**

(*A*) Immunolabeling of TRPV1 in spinal cord of Veh. (5% DMSO+5% Tween80)- or RTX (200 ng/5µl)-treated mice.

(*B*) Quantification of TRPV1 immuno-reactive nerve terminals in superficial layers of spinal cord dorsal horn. ^*^p<0.05 vs. Veh., two tail t-test, n=4-5 mice/group.


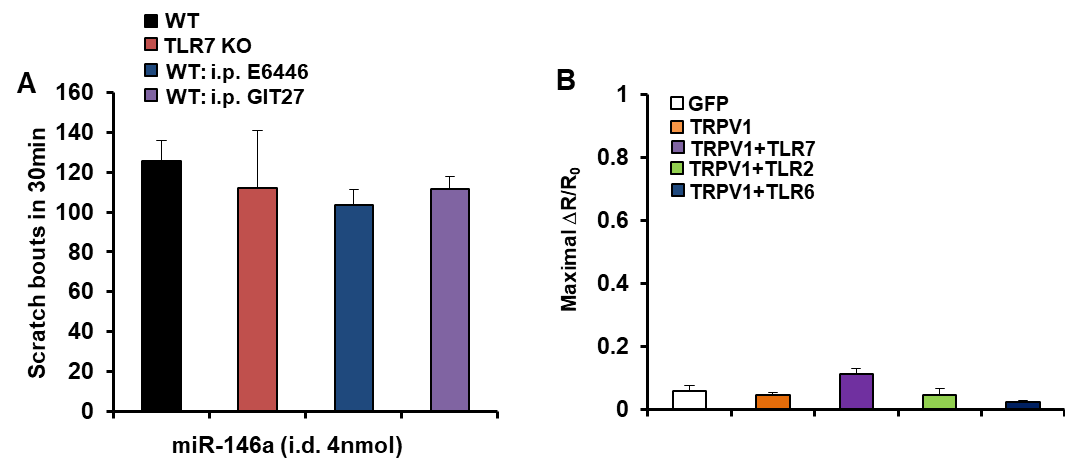


**Supplementary Figure 11. Lack of evidence of direct activation of TRPV1 or TLRs signaling upstream of TRPV1 in response to miR-146a.**

(*A*). Mouse scratching behavior evoked by i.d. injection of miR-146a (4 nmol) was not significantly altered by knockout of Tlr7, i.p. treatment with TLR7/9 inhibitor E6446 or TLR2/6 inhibitor (GIT27) at 10 mg/kg. One-way ANOVA with Tukey’s post-hoc test. N=4-7/group.

(*B*). HEK293 cells transfected with rTRPV1 or co-transfected with rTRPV1 and rTLRs did not show a significant Ca2+ transient upon stimulation with miR-146a at 300 nM when compared to control (GFP-transfected). One-way ANOVA with Tukey’s post-hoc test. N≥260 cells /group.


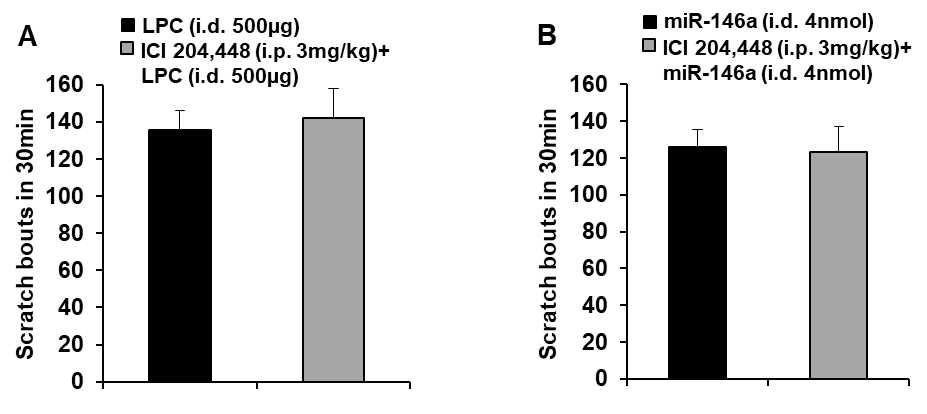


**Supplementary Figure. 12. Kappa-opioid-receptor (KOR) is not involved in LPC- or miR-146a-induced itch.** LPC- (A) or miR-146a (B)-induced scratching behavior was not significantly affected by the KOR selective agonist ICI-204,448. Two-tailed t test, n=13 mice for LPC and 5 for ICI-204,448.


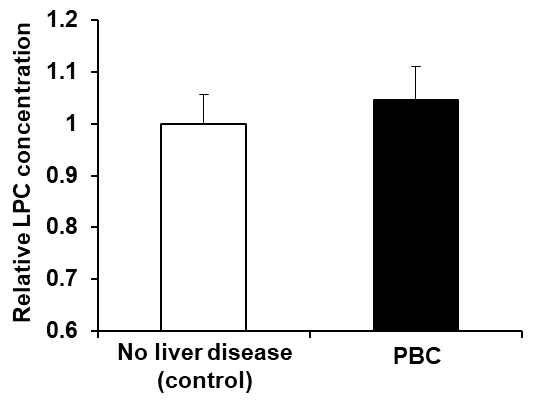


**Supplementary Figure. 13. There was no significant difference in systemic LPC levels between patients in a gastroenterology-hepatology outpatients clinics not having liver disease, as indicated by normal liver histology, referred to as "control" vs PBC patients, the latter with unknown itch/no-itch status.** Two-tailed t test, n=35 for control patients and n=25 for PBC patients, both cohorts recruited via the Duke Gastroenterology-Hepatology outpatient program, non-alcoholic fatty liver disease (NAFLD) clinical research program.

**
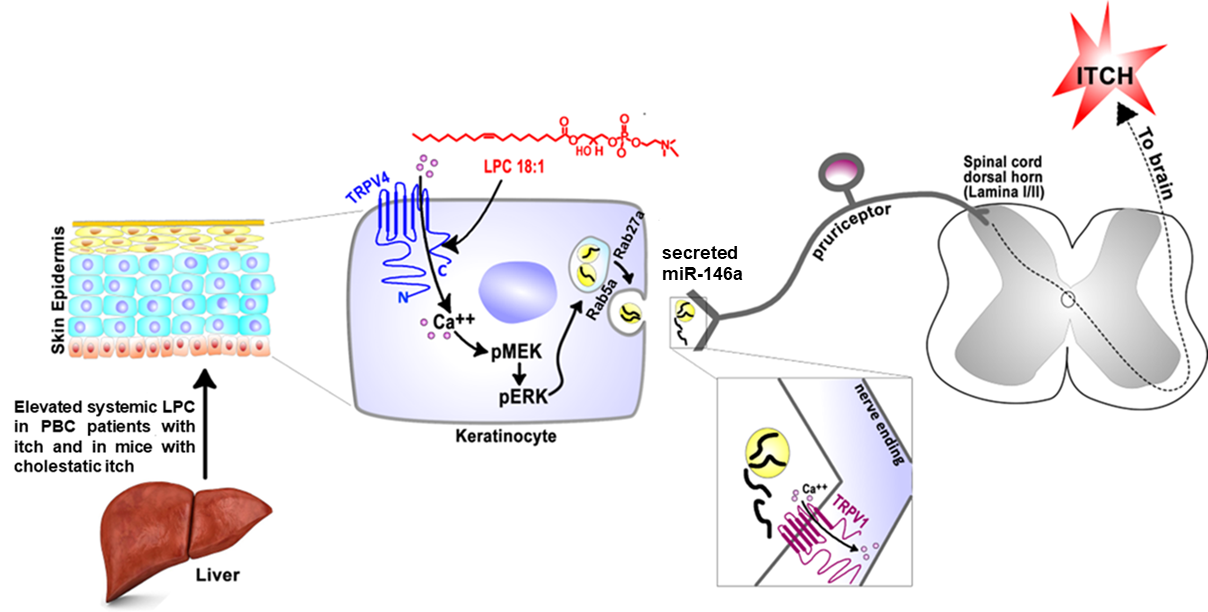
**

**Supplementary Figure 14. Schematic diagram depicting the potential mechanism underlying cholestatic itch.**

Cholestatic liver disease is associated with significantly elevated systemic LPC, which directly activates TRPV4 expressed in skin keratinocytes. This in turn leads to extracellular release of miR-146a via MEK-ERK-Rab5a/Rab27a signaling pathways. miR-146a functions as a pruritogen by activating TRPV1-expressing pruriceptor sensory neurons that innervate the skin. Activation of TRPV1 by miR-146a induces the sensation of itch via central pathways.

**Supplementary Movie 1. 3D structural rendering of TRPV4 tetramer with the green subunit binding of LPC 18:1.**

**Supplementary Movie 2. *Ex vivo* imaging of DRG explants illustrating live Ca^2+^ signal of GCaMP3^+^ sensory neurons in response to miR-146a (300nM) and capsaicin (1µM).**
